# Solute Carrier transporters in tumor metabolism and immune modulation: implications for therapy

**DOI:** 10.1186/s12967-026-07918-4

**Published:** 2026-03-14

**Authors:** Jialin Zhou, Weitao Wen, Ying Xu, Jinming Yu, Dawei Chen

**Affiliations:** Shandong Provincial Key Laboratory of Precision Oncology, Department of Radiation Oncology, Shandong Cancer Hospital and Institute, Shandong First Medical University and Shandong Academy of Medical Sciences, Jinan, Shandong China

**Keywords:** Solute carrier (SLC) transporters, Tumor metabolism, Immune modulation, SLC-targeted strategies

## Abstract

**Background:**

Metabolic reprogramming is a core hallmark of cancer, enabling tumor progression, immune evasion, and therapy resistance. Solute carrier (SLC) transporters, which govern the cellular flux of all nutrients and metabolites, are increasingly recognized as critical drivers of this pathological reprogramming. The altered expression and activity of SLCs in both tumor cells and immune cells profoundly shape the tumor microenvironment (TME) and are directly implicated in clinical outcomes.

**Main Body:**

This review critically examines the roles of key SLC families, particularly those transporting glucose, amino acids, and lipids. We elucidate their dual function in fueling oncogenic proliferation while simultaneously dictating the metabolic competition that leads to the functional suppression of immune cells, including T cells, dendritic cells, and macrophages. We further explore the emerging therapeutic landscape, evaluating strategies that either inhibit SLCs to starve tumors or leverage them to enhance drug delivery and mitigate treatment-related toxicities.

**Conclusion:**

Targeting SLC-mediated metabolic pathways represents a novel and highly promising therapeutic axis in oncology. We conclude by outlining the key translational challenges and future opportunities for SLC-targeted strategies. These approaches hold the potential to overcome immunotherapy resistance, reprogram the immunosuppressive TME, and exploit fundamental metabolic dependencies to improve patient outcomes.

**Supplementary Information:**

The online version contains supplementary material available at 10.1186/s12967-026-07918-4.

## Background

The solute carrier (SLC) superfamily, the largest group of membrane transporters, comprises over 400 members across 65 subfamilies. Characterized by multiple transmembrane α-helices, SLC transporters are classified into distinct structural folds, including the major facilitator superfamily (MFS) fold, leucine transporter (LeuT) fold, and mitochondrial carrier fold. This structural diversity enables SLCs to mediate both passive and secondary active transport, which are essential for maintaining cellular homeostasis [1], [2].

In cancer, SLC transporters are critical regulators of metabolic reprogramming, a hallmark of tumor progression [3]. Tumor cells alter their metabolic pathways to meet the demands of rapid growth, with SLCs facilitating the uptake of key nutrients such as glucose, amino acids, and lipids [4–6]. These transporters not only sustain the metabolic needs of cancer cells but also confer a survival advantage by adapting to tumor microenvironment (TME) [7].

Beyond tumor cell, SLC transporters are instrumental in regulating the function of immune cells within the TME. T cells [8], dendritic cells (DCs) [9], and tumor associated macrophages (TAMs) rely on SLC-mediated nutrient transport for energy production and activation. Stromal cells, including cancer-associated fibroblasts (CAFs) and endothelial cells, also depend on these transporters for their metabolic adaptation, further supporting tumor progression. By modulating nutrient availability and cellular interactions, SLC transporters influence the metabolic dynamics of the TME, shaping both immune responses and tumor progression [10].

While prior reviews have explored the roles of SLC transporters in cancer metabolism and immunity, a comprehensive and integrative analysis of their diverse functions remains lacking. This review aims to address this gap by providing an in-depth examination of SLC transporters in cancer, with a focus on their roles in metabolic reprogramming, immune cell regulation within the TME, and contributions to therapeutic resistance and treatment-related toxicities. By emphasizing the underlying molecular mechanisms, this review offers insights into how targeting SLC transporters can enhance the precision of cancer therapies.

## Main text

### SLC-Driven metabolic rewiring in tumor cells

**Fig. 1:**
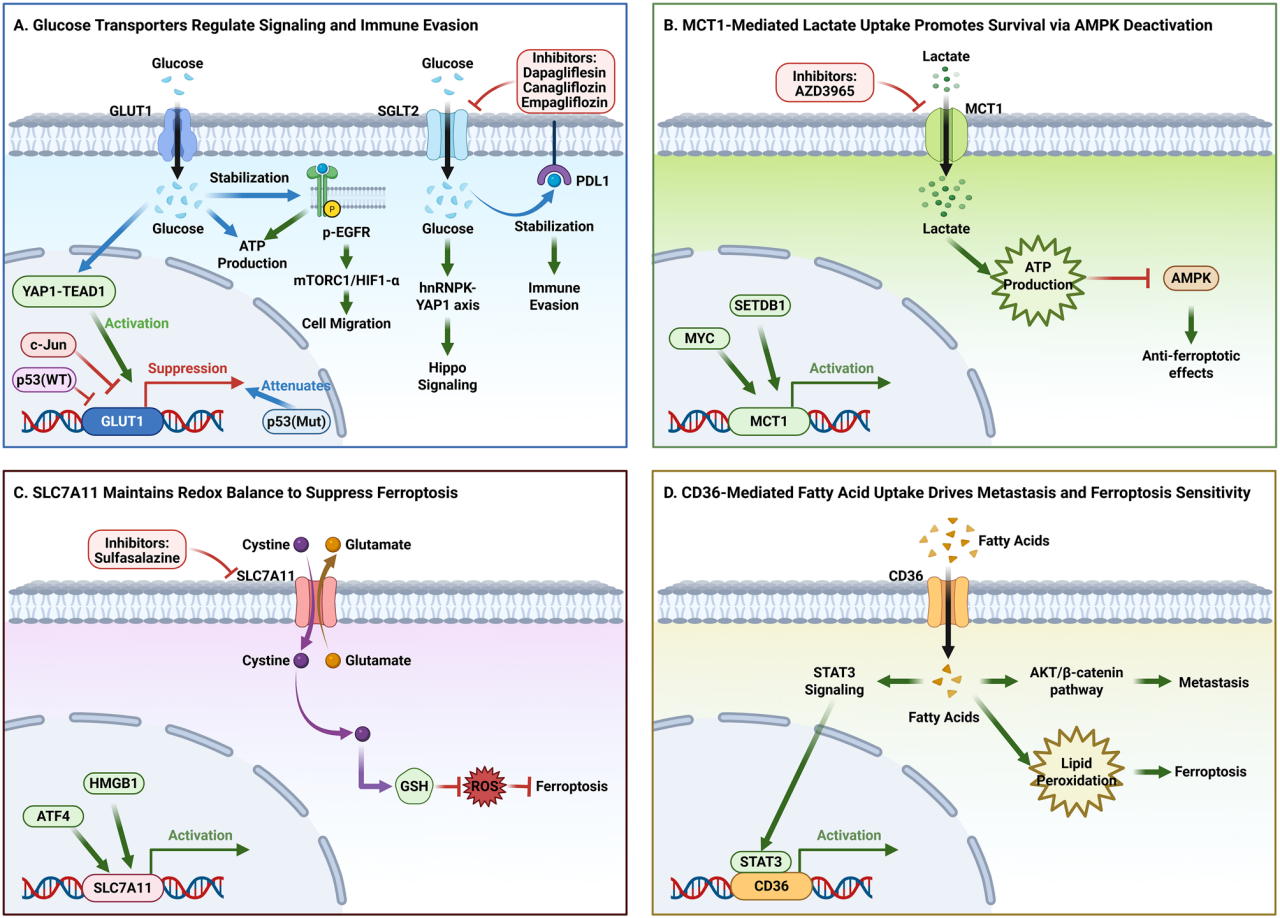
Core SLC transporters in cancer metabolism and progression. (**A-D**) Schematic overview of key SLC-mediated pathways. Arrows denote promotion, transport, or activation; blunt ends denote inhibition. (**A**) Glucose transporters. SGLT2 and GLUT1 facilitate glucose uptake. SGLT2 stabilizes PD-L1 and activates Hippo signaling via hnRNPK-YAP1. GLUT1 expression is regulated by YAP1 (promotion) and c-Jun/p53 (repression). Clinically used SGLT2 inhibitors are shown. (**B**) Lactate transporter MCT1. MCT1 imports lactate, supporting ATP production and AMPK inactivation, thereby inhibiting ferroptosis. Its expression is driven by MYC and SETDB1. The inhibitor AZD3965 is indicated. (**C**) Cystine/glutamate antiporter xCT. xCT imports cystine for glutathione synthesis, suppressing ferroptosis. Its transcription is regulated by ATF4 and HMGA1. Sulfasalazine is a known inhibitor. (**D**) Fatty acid transporter CD36. CD36-mediated fatty acid uptake engages in a feedback loop with STAT3, promotes metastasis via the PI3K-AKT-β-catenin axis, and contributes to lipid peroxidation-associated ferroptosis

Metabolic reprogramming is a hallmark of cancer, characterized by alterations in glucose, amino acid, and lipid metabolism. Cancer cells adapt their metabolism to support uncontrolled growth and survival in nutrient-limited environments [11]. This includes the Warburg effect, where glycolysis predominates even in the presence of oxygen, and the reliance on amino acids, particularly glutamine, which contributes 30–50% of cellular carbon [12]. These metabolic changes enable cancer cells to generate energy and biosynthetic materials, facilitating aggressive growth and survival in hostile microenvironments [13]. As illustrated in Fig. 1, SLC transporters play a critical role in this process by mediating the selective transport of key metabolites across cellular membranes [14]. These transporters can be categorized into five major categories: glucose transporters (SLC2/SLC5), which sustain aerobic glycolysis [15, 16]; amino acid transporters (SLC1/SLC7/SLC38), which enable nutrient uptake [17]; lipid transporters (SLC27), which maintain membrane integrity, neurotransmitter transporters, which modulate cancer progression [18]; and drug transporters (SLC28/SLC29), which affect therapeutic outcomes [19]. This section reviews how these transporter families orchestrate tumor metabolism and contribute to cancer development, progression, and treatment responses, with a focus on their roles in metabolic reprogramming and potential therapeutic implications.

### Coordinated nutrient influx sustains anabolic growth

Coordinated nutrient influx constitutes a fundamental requirement for sustained anabolic growth in cancer. Rather than acting as isolated conduits, SLC transporters form an integrated metabolic infrastructure that couples extracellular nutrient availability to intracellular biosynthetic demand and signaling control. Through coordinated regulation of glucose, amino acid, lipid, and ion fluxes, these transport systems collectively support proliferation, survival, and adaptation to metabolic stress. Nutrient influx pathways are functionally coupled across space and time; oncogenic signaling programs such as MYC, mTOR, and HIF-1α synchronously regulate multiple SLC classes; and SLC-mediated ion homeostasis provides essential cofactors that support global metabolic flux.

Facilitative glucose transporters of the SLC2 family govern the primary entry of carbon into central metabolism. Comparative expression analyses indicate that GLUT1 (SLC2A1) and GLUT3 (SLC2A3) are preferentially enriched in malignant tissues, while GLUT1–4 remain broadly detectable in non-malignant counterparts [20]. Functionally, GLUT1 serves as a dominant high-capacity transporter under hypoxic or nutrient-restricted conditions, sustaining glucose influx when metabolic demand exceeds supply [21]. Although early studies suggested that GLUT1 was largely absent from normal epithelia, subsequent work demonstrated that its upregulation occurs at early stages of mammary tumorigenesis and is also detectable in benign lesions such as thyroid papillary carcinoma [22–24].

GLUT1 expression and activity are reinforced by multilayered regulatory circuits that span chromatin organization, transcriptional control, and post-translational stabilization. Aberrant super-enhancer activation selectively drives SLC2A1 expression in lung adenocarcinoma, linking higher-order genome architecture to oncogenic glucose uptake [25]. Transcriptionally, YAP1–TEAD1 complexes directly induce SLC2A1 to enforce glycolytic phenotypes in breast cancer cells [26], while FOXM1 activation downstream of BTF3 enhances GLUT1 expression in hepatocellular carcinoma [27]. In parallel, METTL5-stabilized c-Myc signaling couples cell-cycle progression to glucose acquisition by sustaining GLUT1 transcription [28]. In addition to protein-coding regulators, an m6A-dependent positive feedback loop between the glycolytic lncRNA SLC2A1-DT and c-Myc has been identified in hepatocellular carcinoma (HCC), further amplifying GLUT1 expression and linking epitranscriptomic control to oncogenic transcriptional programs [29]. Tumor-suppressive constraints also operate at this level, as wild-type p53 represses GLUT1 and GLUT4 in a tissue-dependent manner, a brake that is relieved upon p53 mutation [30]. At the level of membrane trafficking, Akt signaling promotes GLUT1 plasma membrane localization by inhibiting TXNIP-mediated endocytosis [31, 32]. Tumor initiator CD133 further upregulates GLUT1 expression through the HER3/Akt/mTOR pathway [33].

Post-transcriptional and post-translational mechanisms consolidate GLUT1 stability and activity. METTL3-mediated m6A deposition stabilizes SLC2A1 transcripts through IGF2BP-dependent recognition, sustaining glycolysis and tumor growth in colorectal cancer (CRC) [34]. Lipid modification provides an additional layer of control, as S-palmitoylation regulates membrane localization and stability of GLUT1 in glioblastoma [35, 36]. Protein stability is further enhanced by galanin-induced SUMOylation, which protects GLUT1 from ubiquitin–proteasome–mediated degradation [37]. Conversely, intrinsic metabolic restraints persist in specific contexts, where cytoglobin (CYGB) epigenetically silences GLUT1 and hexokinase 2, and c-Jun directly represses SLC2A1 transcription through promoter binding [38, 39]. Functionally, deregulated GLUT1 drives multiple malignant phenotypes. In gastric cancer (GC), elevated GLUT1 promotes tumor cell proliferation and metastatic dissemination [40], whereas in bladder cancer, GLUT1 enhances cell migration through a GLUT1–mTORC1–HIF-1α–PKM2 positive feedback loop [41]. Systemic metabolic states can further amplify this axis, as type 2 diabetes enhances GLUT1-mediated glucose uptake to accelerate breast cancer progression [42], while Fusobacterium nucleatum–induced lactate production fuels oral squamous cell carcinoma growth via GLUT1-dependent pathways [43]. Consistent with its central role, pharmacological inhibition of GLUT1 using oxabicycloheptene sulfonate (OBHS) suppresses breast cancer growth by attenuating PI3K–Akt signaling [44].

Downstream of GLUT1-dominated glucose influx, additional facilitative glucose transporters provide context-dependent reinforcement of carbon acquisition under environmental and therapeutic stress. Under hypoxic conditions, HIF-1α induces GLUT2 (SLC2A2) expression, augmenting glucose uptake in oxygen-limited environments [45]. In KRAS-mutant cancers, a p38γ–PFKFB3 axis transcriptionally activates GLUT2 to reinforce glycolytic flux and pancreatic tumor growth [46].

In contrast, GLUT3 emerges as a stress-specialized transporter that becomes indispensable under therapy-induced metabolic pressure. Although secondary to GLUT1 under basal conditions, the high glucose affinity of GLUT3 enables cancer cells to maintain energy homeostasis in therapy-resistant states. In tyrosine kinase inhibitor (TKI)–resistant tumors, a Cav1–GLUT3 axis preserves glucose uptake despite reduced extracellular glucose availability [47, 48]. Multiple regulatory inputs converge on GLUT3, including AMPK/CREB1 signaling [49], RIP140 [50], ZEB1 [51], and YTHDC1 [52]. Beyond glucose transport, GLUT3 also mediates vitamin C uptake in acute myeloid leukemia (AML), linking transporter activity to redox regulation [53]. In glioblastoma, activation of an ATF4–DDIT4–GLUT3 axis underlies resistance to temozolomide (TMZ) therapy [54].

GLUT4 (SLC2A4) occupies a distinct niche within this hierarchy, exhibiting context-dependent and sometimes paradoxical roles [5]. Although upregulated in multiple tumor types, reduced GLUT4 expression correlates with favorable prognosis in breast cancer [55]. Its regulation integrates epigenetic and transcriptional inputs, including DNA hypermethylation [55], and control by KLF8 and ISL1 [56, 57]. Under low-glucose conditions, the AMPKα2/HNF4A/BORIS/GLUT4 pathway enhances invasion and metastasis in HCC [58]. Post-transcriptionally, ALKBH5-mediated m6A demethylation of GLUT4 contributes to resistance to HER2-targeted therapies [59], whereas GLUT4 inhibition suppresses proliferation in enzalutamide-resistant prostate cancer cells [60].

Complementing facilitative glucose influx, monocarboxylate transport via MCT family members redistributes metabolic burden by coupling lactate exchange to cellular redox balance. MCT1 (SLC16A1) functions as the dominant lactate transporter in retinoblastoma, where its disruption impairs energy metabolism and cell viability [61, 62]. In multiple myeloma, elevated MCT1 expression correlates with response to lenalidomide maintenance therapy [63]. Epigenetically, SETDB1 enhances MCT1 expression through K473 methylation to promote lactate shuttling and tumor progression [64]. Conversely, hyperoxic conditions suppress the MYC–MCT1 axis, leading to intracellular lactate accumulation and acidification that restrains tumor growth and metastasis [65]. In melanoma, high MCT1 expression marks a subpopulation with enhanced metastatic competence [66]. Pharmacological targeting of MCT1 with AZD3965 suppresses tumor growth in vivo [62]. MCT4 (SLC16A3) fulfills a complementary role by mediating lactate export in highly glycolytic tumors, thereby sustaining intracellular redox balance. Elevated MCT4 expression independently predicts poor overall survival in bladder cancer [67]. Mechanistically, CD147 K234me2 promotes CD147–MCT4 interaction and membrane trafficking in non-small cell lung cancer (NSCLC) cells [68]. Additionally, SYVN1-mediated ubiquitination ensures MCT4 localization at the membrane, supporting lung adenocarcinoma progression [69]. At the post-transcriptional level, circ235 upregulates MCT4 expression by sponging miR-330-5p, further reinforcing glycolysis and tumor growth [70]. MCT4 overexpression is commonly detected in circulating tumor cells (CTCs) from NSCLC patients, indicating its role in the metastatic dissemination and treatment resistance [71].

Under conditions notably characterized by nutrient scarcity, sodium-dependent glucose transporters provide an additional scavenging layer. SGLT1 (SLC5A1) exhibits cancer-type–specific prognostic relevance, associating with poor outcomes in breast cancer [72], while correlating with improved prognosis in pancreatic ductal adenocarcinoma (PDAC) [73]. Functional ablation of SGLT1 suppresses proliferation, induces apoptosis, and reprograms cellular metabolism in gastric cancer (GC), supporting its role as an adaptive glucose acquisition mechanism under low-glucose stress [74]. The hierarchical and context-dependent activities of facilitative and sodium-dependent glucose transporters, coupled with lactate shuttling via MCTs, ensure a robust and adaptable carbon supply that sustains glycolytic flux and redox balance under hypoxia, oncogenic signaling, and therapeutic stress.

Amino acid acquisition constitutes a second major anabolic axis that operates downstream of glucose-driven carbon influx and supports both biosynthetic demand and signaling fidelity. Systematic analyses have identified 68 amino acid transporters across 13 SLC subfamilies as key mediators of amino acid flux [75]. Among these, SLC1A3 (EAAT1) functions as a glutamate–aspartate antiporter that safeguards intracellular aspartate availability under nutrient stress. In T-cell acute lymphoblastic leukemia, EAAT1 sustains nucleotide biosynthesis and leukemic proliferation by maintaining cytosolic aspartate pools through glutamate–aspartate exchange [76]. Importantly, p53 upregulates SLC1A3 expression, enabling aspartate utilization and cellular survival under glutamine-depleted conditions in hypoxic TME and xenograft models [77]. Furthermore, EAAT1 further buffers metabolic stress imposed by asparaginase treatment by maintaining coordinated aspartate, glutamate, and glutamine metabolism, thereby supporting tumor growth and metastatic progression [78, 79].

Glutamine uptake represents a dominant entry point into amino acid metabolism and is primarily mediated by ASCT2 (SLC1A5). Beyond its role in fueling bioenergetic and biosynthetic processes [80], ASCT2 actively promotes tumor vascularization and epithelial–mesenchymal transition, linking nutrient influx to invasive behavior [81]. Its oncogenic function has been demonstrated across multiple tumor types, including head and neck squamous cell carcinoma (HNSCC) [82], glioma [83], and CRC, where a super-enhancer–associated lncRNA cascade amplifies SLC1A5 expression [84]. In lung cancer, MYC-driven SLC1A5 induction establishes glutamine addiction and facilitates metastatic progression [85]. In multiple myeloma, SLC1A5 expression correlates with advanced disease stages and resistance to proteasome inhibitors (PIs) [86]. Consistently, disruption of this axis through inspiratory hyperoxia suppresses MYC/SLC1A5-dependent metastasis in lung cancer, highlighting a therapeutically exploitable vulnerability [85].

Beyond single transporters, amino acid influx is coordinated through transporter hubs. CD98 (SLC3A2) functions as a central scaffold for large neutral amino acid and polyamine transport. Oncogenic anaplastic lymphoma kinase (ALK) signaling stabilizes SLC3A2 via MARCH11 to promote tumor growth [87], while short-term acidosis induces its ubiquitination and degradation through ZFAND5, suppressing tumor growth [88]. In HCC, ALDH2 inhibits BSG expression via the TGF-β1 pathway, indirectly suppressing SLC3A2 and reducing hepatocarcinogenesis [89]. Consistent with its oncogenic roles, targeting SLC3A2-mediated polyamine uptake significantly suppresses tumor progression and prolongs survival in neuroblastoma models [90], while in lung and liver cancers, SLC3A2 promotes proliferation through KRAS and TGF-β1 signaling pathways, respectively [89, 91].

Cationic amino acid transport further reinforces anabolic signaling. In glioblastoma stem cells (GSCs), upregulation of SLC7A2 (CAT2) drives lysine catabolism and supports metabolic reprogramming associated with stemness [92]. In PDAC, RIOK3 enhances arginine uptake through SLC7A2 to activate mTORC1 signaling and promote invasion and metastasis [93]. SLC7A3 (CAT3) mediates extracellular arginine uptake and, while dispensable for normal hematopoiesis and leukemogenesis [94], SP1-driven SLC7A3 expression in osteosarcoma enhances arginine uptake and cell migration through a self-reinforcing SP1 stabilization loop [95].

Essential amino acid transport is predominantly mediated by LAT1 (SLC7A5), which facilitates uptake of large neutral amino acids such as leucine, isoleucine, valine, phenylalanine, tyrosine, tryptophan, and methionine [96, 97]. LAT1 expression integrates stress-responsive and oncogenic inputs, being transcriptionally regulated by ATF4 and mutant p53 [98, 99]. In GC, a circARID1A–IGF2BP3–SLC7A5 complex promotes tumor proliferation [100], while in CRC, LAT1 sustains amino acid availability downstream of KRAS activation to drive tumorigenesis [101]. In estrogen receptor–positive breast cancer, estrogen-induced SCRIB assembles LAT1–SLC3A2 complexes at the plasma membrane to ensure efficient leucine uptake and sensitivity to tamoxifen therapy [102].

By contrast, LAT2 (SLC7A8) exhibits context-dependent behavior, promoting tumor growth in basal cell carcinoma [103, 104], while paradoxically suppressing growth and metastasis in lung adenocarcinoma [105].

Complementing these systems, SLC38A2 (SNAT2) mediates adaptive glutamine uptake in response to microenvironmental cues. CAF-derived lncRNA LINC01614 induces SLC38A2 and SLC7A5 expression in lung adenocarcinoma to enhance glutamine influx [106], while miR-10b-5p–dependent regulation links SLC38A2 activity to liver cancer progression [107]. Elevated SLC38A2 expression supports essential amino acid availability and lactate accumulation, contributing to tumor cell survival [108].

Lipid transport constitutes a parallel anabolic axis that supports membrane biogenesis, signaling, and metastatic competence. Members of the SLC27 family facilitate fatty acid uptake in a context-dependent manner. In breast cancer, SLC27A4 enhances fatty acid influx to promote cell-cycle progression, epithelial–mesenchymal transition, and invasive growth [109]. In bladder cancer, FATP2 (SLC27A2) couples fatty acid metabolic inputs to PI3K–Akt–mTOR signaling to support proliferation and migration. Pharmacological inhibition of FATP2 induces ATF3 expression, suppresses PI3K–Akt–mTOR activation, and triggers cell-cycle arrest and apoptosis [110], while FATP5 correlates with improved prognosis in CRC [111].

CD36 represents a multifunctional lipid transporter whose oncogenic impact is shaped by metabolic context. In GC, fatty acid availability enhances CD36 expression and activity through O-GlcNAcylation-dependent mechanisms. Elevated O-GlcNAcylation activates NF-κB–driven CD36 transcription and directly modifies CD36 to increase fatty acid uptake, collectively promoting tumor cell invasion and metastasis [112]. Palmitic acid promotes tumor metastasis through CD36-dependent activation of the AKT/GSK-3β/β-catenin axis. CD36-mediated fatty acid sensing activates AKT, leading to inhibitory phosphorylation of GSK-3β and nuclear accumulation of β-catenin, thereby enhancing migratory and invasive programs. Clinically, elevated CD36 expression correlates with poor prognosis, underscoring its role in lipid-driven metastatic progression [113]. Similar lipid-driven oncogenic signaling is observed in cervical cancer, where dietary oleic acid induces CD36 to activate Src–ERK pathways, and in HCC, where CD36 reprograms metabolism via Src–PI3K–AKT–mTOR signaling to enhance aerobic glycolysis and lactate production [114, 115]. Conversely, in CRC, CD36 interacts with glypican 4 to suppress β-catenin–c-Myc signaling and inhibit aerobic glycolysis, revealing a tumor-suppressive role in specific settings [116]. Nevertheless, high CD36 expression is broadly associated with adverse prognosis and therapeutic resistance, including early-stage HER2-positive breast cancer where it predicts resistance to trastuzumab-based therapy [117–119]. CD36 expression signatures also hold immunological relevance, as the CD36–BATF2/MYB axis predicts response to anti–PD-1 immunotherapy in GC [120]. In hematological malignancies, CD36-dependent lipid metabolism promotes immune escape and resistance to hypomethylating agents in AML. Inhibition of CD36 using statins or the small-molecule inhibitor SMS121 sensitizes leukemia cells to decitabine and cytarabine by disrupting fatty acid oxidation–oxidative phosphorylation (OXPHOS) programs [121–123].

Beyond macronutrient transport, ion homeostasis provides an additional layer of metabolic control. The zinc transporter SLC39A6 (LIV-1) regulates intracellular Zn^2 +^ levels and promotes cancer progression [124, 125]. Overexpression of SLC39A6 enhances the expression of metastasis-associated genes, including MMP1, MMP3, MYC, and SLUG, thereby increasing metastatic potential in vivo [126]. In liver cancer, the SLC39A6–CREB1 axis suppresses mitochondrial function through PCK1 modulation [127]. Genome-wide association studies further associate SLC39A6 variants with survival in esophageal squamous-cell carcinoma, while LIV-1 overexpression predicts poor prognosis in breast cancer, particularly triple-negative subtypes [128–131]. These observations have spurred translational efforts targeting LIV-1 using antibody–drug conjugates such as SGN-LIV1A, as well as its development as a prognostic biomarker in gastric adenocarcinoma [129, 132–134]. Iron homeostasis constitutes another critical ionic axis in cancer metabolism [135]. The divalent metal transporter SLC11A2 (DMT1) mediates non-heme iron uptake and contributes to CRC progression, where it links iron uptake with JAK-STAT3 signaling [136]. At the subcellular level, DMT1 regulates mitochondrial iron translocation, which influences metastatic growth [137]. Overexpression of DMT1 is associated with metastatic adenocarcinoma, and targeting DMT1 with compounds like pyrimidinone 8 inhibits its transport activity, offering a potential therapeutic strategy [138, 139].

Magnesium transport further illustrates the integration of ionic balance with anabolic signaling. SLC41A1 functions as a tumor-suppressive magnesium exporter in PDAC, where Mg^2 +^ efflux attenuates Akt–mTOR signaling, induces Bax expression, and triggers mitochondrial depolarization and apoptosis [140]. Complementing this mechanism, phosphatases of regenerating liver (PRLs) regulate cellular Mg^2 +^ homeostasis through non-catalytic interactions with CNNM ion transporters, restraining Mg^2 +^ efflux and thereby promoting tumor growth via a noncanonical PRL–CNNM signaling axis [141]. In renal cell carcinoma, lncRNA-mediated cis-activation of SLC47A2 further underscores the contribution of ion transporter regulation to tumor biology [142]. Unexpectedly, transporters traditionally associated with neurotransmission can also be co-opted by cancer cells. The dopamine transporter SLC6A3 (DAT) is aberrantly expressed in clear cell renal cell carcinoma, where it serves as a potential diagnostic and prognostic biomarker [143, 144].

#### Metabolic plasticity enables adaptation to microenvironmental stress

Cancer cells rely on metabolic plasticity to survive fluctuating and hostile microenvironments. Across tumor types, SLC rewiring converges on several conserved adaptive outputs, including maintenance of anabolic signaling, reinforcement of epigenetic programs, preservation of nutrient sensing, and buffering of stress-induced metabolic damage.

A central adaptive output under therapeutic pressure is the reinforcement of anabolic signaling and epigenetic feedback loops that sustain treatment resistance. GLUT1 has been implicated in resistance to androgen deprivation in castration-resistant prostate cancer and contributes to chemoresistance in pancreatic cancer [145, 146].

Dysregulation of the miR-148a–GLUT1 axis further drives chemoresistance in intrahepatic cholangiocarcinoma, while autophagy-dependent GLUT1 signaling mediates tamoxifen resistance in breast cancer [147, 148]. Radiotherapy similarly reshapes glucose flux, as irradiation induces GLUT3 translocation to the plasma membrane to support metabolic reprogramming [149]. Epigenetic regulation further integrates transporter control with drug response. The deacylase SIRT7 represses GLUT3 expression by binding its enhancer and removing H3K122 succinylation, thereby limiting gemcitabine uptake and promoting chemoresistance in pancreatic cancer [150]. Therapy resistance also involves metabolic efflux and sodium–glucose transport, as elevated MCT4 predicts poor response to anti–PD-1 therapy in HCC [151]. Moreover, SGLT1 is implicated in treatment resistance. The PKCδ/EGFR axis-mediated upregulation of SGLT1 contributes to acquired resistance to EGFR TKIs [152].

Amino-acid transport provides a complementary mechanism linking nutrient uptake to mTOR activation and chromatin remodeling. In lung cancer, IGF2BP2 stabilization of SLC7A5 enhances methionine uptake and activates AKT–mTOR signaling, while SAM-dependent H3K4 trimethylation establishes a self-reinforcing IGF2BP2–SLC7A5 circuit that promotes radioresistance [153]. Similarly, LAT2-driven glutamine uptake activates mTOR signaling and reinforces glutamine-dependent metabolic feedback loops, sustaining glycolysis and promoting gemcitabine resistance in pancreatic cancer [154]. Longitudinal multi-omics analyses of esophageal squamous cell carcinoma have identified LAT2 as a novel multidrug resistance gene. Acquired SLC7A8 mutations, together with dynamic promoter hypomethylation of metabolic transport pathways, drive clonal expansion and therapeutic resistance during chemotherapy [155].

Nutrient limitation and hypoxia activate a second conserved adaptive program centered on preservation of nutrient sensing and biosynthetic capacity. Under hypoxia, SLC38A2 upregulation promotes endocrine resistance in breast cancer [156], while in triple-negative breast cancer (TNBC), its upregulation correlates with glutamine dependence and oxidative stress resistance, predicting poor prognosis [157]. In HCC, hypoxia suppresses HNF4A-dependent SLC25A15 expression, triggering glutamine metabolic reprogramming via SLC1A5 upregulation and OGDHL inhibition to fuel lipid synthesis and tumor progression. This rewired glutamine dependency also creates a therapeutic vulnerability that enhances sensitivity to glutamine-targeted strategies and immune checkpoint blockade (ICB) [158]. Glucose starvation reveals a complementary lysosomal nutrient-sensing adaptation. In renal cancer, O-GlcNAcylation stabilizes TFE3 and induces the lysosomal amino-acid transporter SLC36A1, sustaining mTOR activity during glucose deprivation [159]. Consistently, SLC36A1 upregulation driven by RB loss or enhanced translation reactivates mTORC1 signaling and underlies acquired resistance to CDK4/6 inhibitors in melanoma [160].

Physical stress imposes a distinct metabolic constraint that is resolved through lipid homeostasis. During matrix detachment, cancer cells experience ER stress–associated lipid saturation due to reduced SCD1 activity. CD36 is induced in an AMPK- and p38-dependent manner to selectively import monounsaturated fatty acids (MUFAs), thereby buffering saturated fatty acid–induced lipotoxicity. This adaptive lipid remodeling preserves membrane homeostasis and supports survival and metastatic competence under detachment-associated stress [161].

Across these diverse stress contexts, epigenetic and clonal mechanisms recurrently shape transporter-mediated adaptation, with DNA methylation emerging as a pervasive determinant of SLC expression programs linked to metabolic plasticity, immune responsiveness, and therapeutic sensitivity [162].

#### Redox control and ferroptosis resistance as metabolic checkpoints

Ferroptosis represents a lipid peroxidation–driven form of regulated cell death that is tightly constrained by cellular redox balance. Emerging evidence indicates that SLC transporters function as central metabolic checkpoints that define ferroptosis susceptibility by integrating lactate flux, lipid composition, and antioxidant capacity. In HCC, MCT1-mediated lactate uptake enhances ATP production and suppresses AMPK activation [163]. In ovarian cancer, curcumin derivative NL01 induces ferroptosis through HCAR1/MCT1 signaling [164]. Beyond lactate, lipid uptake critically influences ferroptosis sensitivity. In HCC, SLC27A4 selectively enhances monounsaturated fatty acid uptake, remodeling membrane phospholipids to suppress lipid peroxidation and confer resistance to sorafenib-induced ferroptosis [165].

The cystine/glutamate antiporter SLC7A11 (xCT), in complex with SLC3A2 forming system Xc^−^, functions as a primary gatekeeper of cystine availability for redox homeostasis, yet its activity is further shaped by a multilayered regulatory network.

In parallel, post-transcriptional mechanisms involving microRNAs including miR-5096, ubiquitin-associated regulators such as TRIM3, and RNA-binding proteins like NKAP modulate SLC7A11 mRNA stability, splicing, or turnover, thereby fine-tuning ferroptosis sensitivity across tumor contexts [166–168]. Tumor–stromal communication further reinforces this regulatory layer, as CAF-derived exosomal ROR1–AS1 enhances SLC7A11 expression in lung cancer cells [169].

Epitranscriptomic regulation constitutes an additional layer of SLC7A11 control that links chromatin state to mRNA stability and ferroptosis sensitivity. m6A erasers such as ALKBH5 destabilize SLC7A11 transcripts and sensitize CRC and NSCLC to ferroptosis [170, 171]. PRMT5-mediated histone modifications repress ALKBH5 transcription, indirectly reinforcing SLC7A11 expression and establishing an epigenetic–epitranscriptomic axis that promotes ferroptosis resistance [172, 173].

Beyond transcript-level regulation, SLC7A11 protein abundance is dynamically governed by ubiquitination–deubiquitination cycles and stress-responsive post-translational modifications. Deubiquitinases including OTUB1 and USP52 directly stabilize SLC7A11 protein [174, 175], while the antagonistic CRL3^KCTD10–USP18 axis fine-tunes its abundance [176]. USP8 indirectly reinforces SLC7A11 stability by stabilizing OGT and promoting O-GlcNAcylation of SLC7A11 which suppresses ubiquitination and ferroptosis across multiple tumor contexts [177]. In contrast, the E3 ligase SOCS2 promotes K48-linked polyubiquitination and proteasomal degradation of SLC7A11, enhancing ferroptosis and therapeutic sensitivity [178]. Phosphorylation-dependent crosstalk hierarchically integrates metabolic stress signaling, as AMPKα1-mediated phosphorylation of ZDHHC8 enhances SLC7A11 palmitoylation and ferroptosis resistance under energy stress conditions [36]. While high SLC7A11 expression supports tumor growth by buffering oxidative stress, it may simultaneously suppress metastatic dissemination by increasing vulnerability to oxidative damage in CTCs [179]. In advanced ovarian cancer, 4EBP1-mediated translational upregulation of SLC7A11 restrains ferroptosis induced by MEK inhibitors [180]. Functionally, the cystine–glutamate antiporter system Xc^−^ represents the terminal execution node of this regulatory network, serving as a central gatekeeper of ferroptosis by maintaining redox homeostasis through cystine–glutamate exchange. Structurally, system Xc^−^ is a heterodimer composed of the catalytic light chain SLC7A11 and the chaperone heavy chain SLC3A2. As an essential subunit of system Xc^−^, SLC3A2 enables the m6A reader YTHDC2 to induce endogenous ferroptosis [181]. Disruption of the SLC3A2–glutathione axis inhibits cystine uptake, depletes glutathione, and inactivates GPX4, leading to lipid peroxidation accumulation and ferroptosis sensitization. This collapse of redox homeostasis overcomes GPX4-driven chemoresistance by disabling intrinsic antioxidant rescue programs [182]. Post-translational modification further modulates this checkpoint, as B3GNT3-mediated N-glycosylation stabilizes SLC3A2 and the system Xc^−^ complex and confers ferroptosis resistance in PDAC [183]. Complementing this regulatory framework, SLC7A11 (xCT) mediates cystine uptake to sustain glutathione synthesis and protect cancer cells from oxidative stress [184].

#### Beyond transport: SLC-Dependent signaling and scaffolding functions

Several SLCs function as membrane-associated signaling adaptors that stabilize receptor activity and amplify downstream pathways. In lung adenocarcinoma, GLUT1 physically associates with phosphorylated EGFR, protecting it from degradation and sustaining EGFR signaling independently of glucose transport, contributing to cancer progression [185]. In TNBC, SGLT1 depletion reduces EGFR phosphorylation and attenuates downstream AKT and ERK signaling [186]. In lung cancer, SLC3A2 forms a fusion with NRG1, generating a membrane-anchored ligand reservoir. KRAS mutations amplify this signaling axis by enhancing ADAM17-mediated cleavage of the fusion protein, which subsequently activates ERBB2–ERBB3 signaling and downstream PI3K/Akt/mTOR pathways, leading to tumor progression and therapeutic resistance [91].

In addition, several SLCs exert oncogenic functions by serving as transport-independent signaling platforms. SGLT2 interacts with hnRNPK to facilitate its nuclear translocation, which enhances hnRNPK-mediated YAP1 transcription and activates the Hippo signaling pathway, thereby ultimately driving pancreatic cancer cell growth and progression both in vitro and in vivo [187]. In HCC, the lncRNA SNHG1 upregulates SLC3A2 to sustain Akt pathway activation, thereby promoting cell survival and sorafenib resistance independently of canonical nutrient transport [188]. In GC, GLUT3 acts as more than a glucose transporter and functions as a metabolic–epigenetic rheostat. By sustaining LDHA expression and lactate-associated metabolic flux, GLUT3 facilitates histone lactylation at H3K9, H3K18, and H3K56, which is closely linked to EMT activation and metastatic potential [189]. Epigenetic reactivation of SLC5A8 restores the uptake of tumor-suppressive metabolites, enabling intracellular inhibition of histone deacetylases and triggering apoptosis, thereby establishing a bidirectional crosstalk between transporter activity and chromatin regulation [190].

### SLC-Mediated immune evasion in the TME

**Fig. 2:**
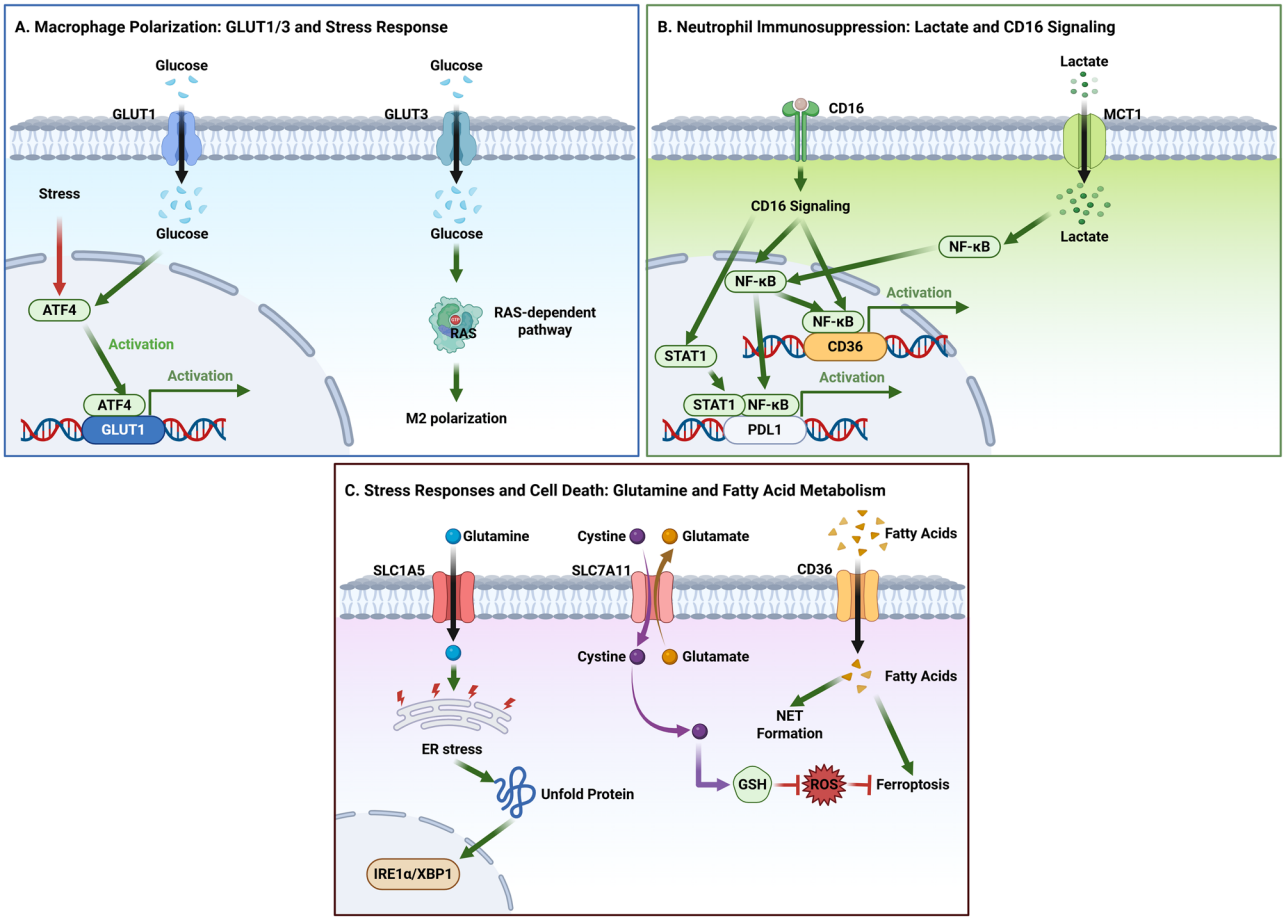
Metabolic reprogramming of myeloid cells by SLC transporters. (**A**) Macrophage polarization. GLUT1 expression is induced under stress (e.g., via ATF4). GLUT3 promotes M2 polarization through a RAS-dependent pathway. (**B**) Neutrophil immunosuppression. Lactate uptake via MCT1 and CD16 signaling converge on NF-κB and STAT1 activation to drive PD-L1 expression. CD16 signaling also upregulates CD36. (**C**) Stress responses and cell death. SLC1A5-mediated glutamine uptake influences ER stress responses (IRE1α/XBP1). The xCT system maintains redox homeostasis, affecting ferroptosis. CD36-mediated fatty acid uptake promotes both NET formation and ferroptosis

**Fig. 3:**
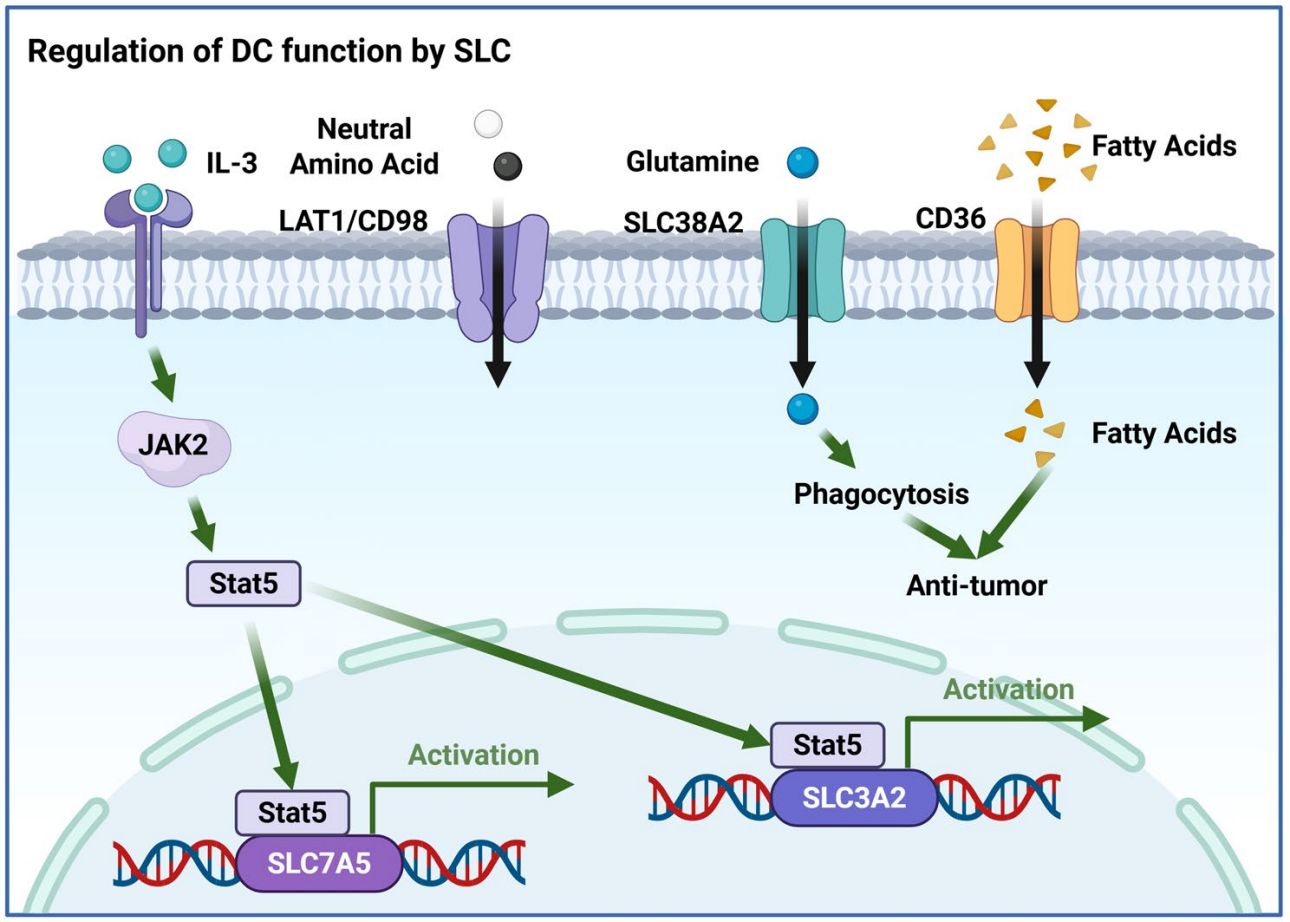
Regulation of DC function by SLC transporters. The cytokine IL-3 activates the JAK2–STAT5 pathway, leading to transcriptional upregulation of amino acid transporters LAT1 and CD98. Glutamine uptake via SLC38A2 and fatty acid uptake via CD36 concurrently modulate DC anti-tumor activity, with CD36 additionally enhancing phagocytic capacity

The TME is a dynamic metabolic ecosystem where immune and stromal cells engage in complex metabolic crosstalk, mediated in part by SLC transporters. These transporters shape the metabolism and function of distinct cell populations, including T cells, macrophages, myeloid-derived suppressor cells (MDSCs), DCs, and stromal cells. Each population exhibits unique metabolic profiles and SLC transporter expression patterns that determine their function within the TME. For instance, as shown in Fig. 2, T cells activation and effector functions are critically dependent on glucose transporters (GLUT1/2/3) and amino acid transporters (SLC7/38 families), where myeloid cells are regulated by fatty acid transporters that modulate their immunosuppressive properties in Fig. 3.

#### Metabolic competition shapes immune cell fitness in the TME

**Fig. 4:**
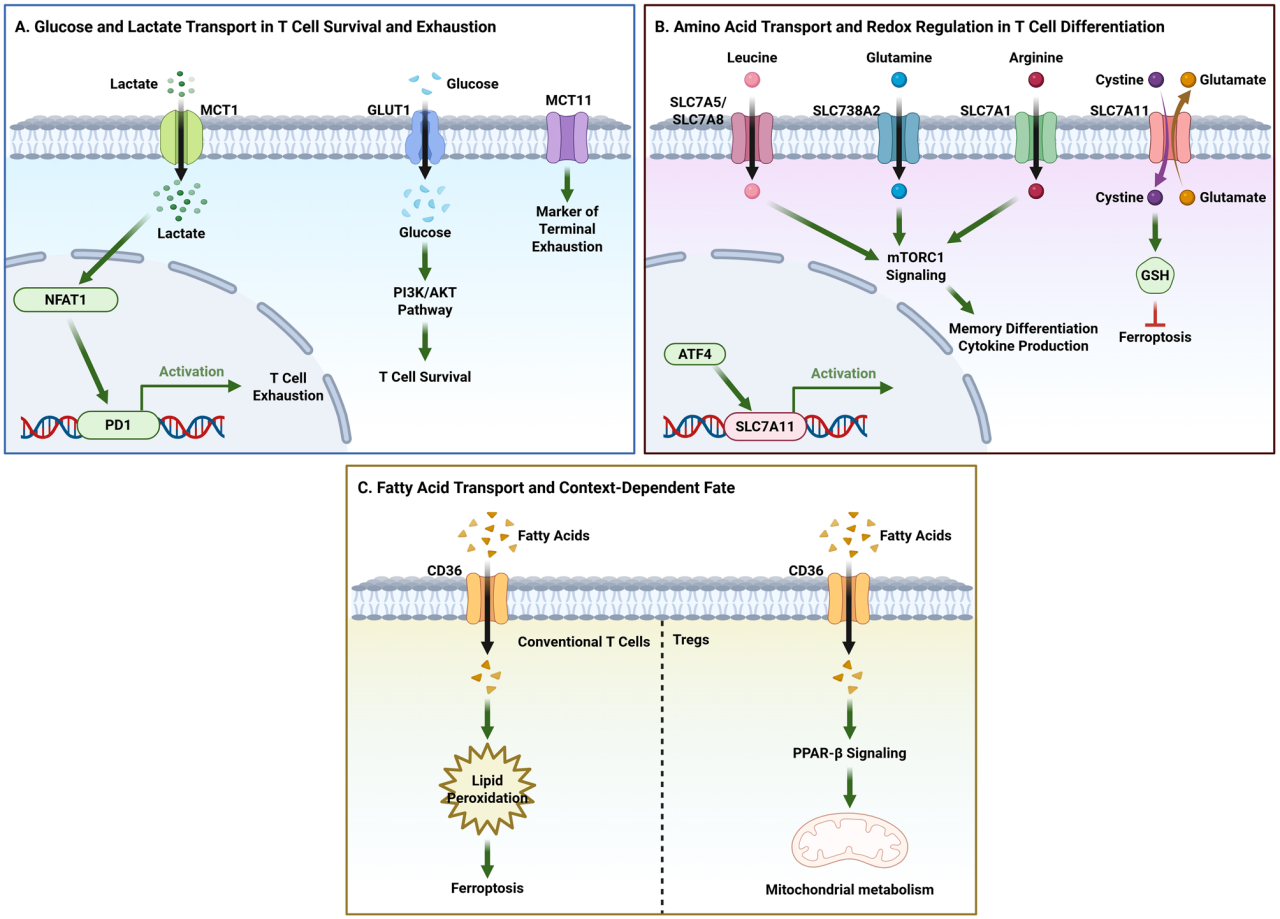
Roles of SLC transporters in determining T cell fate.(**A**) Glucose and lactate transport. GLUT1 supports T cell survival via the PI3K/AKT pathway. MCT1 activity is linked to NFAT1-driven PD-1 expression and the exhausted state. MCT11 (SLC16A11) is a marker of terminal exhaustion. (**B**) Amino acid transport and redox regulation. Leucine (via SLC7A5/SLC7A8), glutamine (via SLC38A2), and arginine (via SLC7A1) transporters converge on mTORC1 signaling to influence memory differentiation and cytokine production. Separately, the SLC7A11 system, transcriptionally regulated by ATF4, maintains glutathione levels to regulate sensitivity to ferroptosis. (**C**) Fatty acid transport. CD36-mediated fatty acid uptake promotes lipid peroxidation and ferroptosis in conventional T cells. In tumor-infiltrating regulatory T cells (Tregs), CD36 engagement enhances mitochondrial metabolism via PPAR-β signaling

Metabolic competition for key nutrients within the tumor microenvironment (TME) represents a fundamental mechanism by which tumors suppress immune cell fitness and effector function, with SLC transporters emerging as central determinants of T cell metabolic fate (Fig. 4). Among distinct metabolic constraints, competition for glucose constitutes a primary and broadly shared limitation across multiple immune compartments. TAMs actively contribute to this competitive landscape by secreting IL-8, which induces GLUT3 expression in tumor cells and enhances glycolytic flux [49, 191]. As a consequence, glucose-restricted conditions impair T cell metabolic programming and antitumor activity. GLUT1 serves as a pivotal regulator of T cell metabolic fitness under such conditions. Enforced GLUT1 expression enhances glucose uptake, sustains glycolytic metabolism, and improves persistence and antitumor efficacy of CAR-T cells [192, 193]. In contrast, GLUT1 deficiency redirects T cell metabolism toward oxidative phosphorylation, resulting in excessive reactive oxygen species (ROS) accumulation and functional exhaustion [194].

Beyond glucose, competition for amino acids represents a second major axis shaping immune competence in the TME, affecting both adaptive and innate immune populations. In colon cancer, tumor cells overexpress the methionine transporter SLC43A2, avidly consuming methionine and depriving CD8^+^ T cells of this essential nutrient. Methionine restriction in T cells reduces intracellular S-adenosylmethionine (SAM) levels, leading to loss of H3K79me2, impaired STAT5 expression, and defective effector differentiation [195]. Amino acid transport also governs immune cell–intrinsic metabolic fitness. Coordinated activity of SLC7A1 and SLC38A2 is required for T cell memory differentiation through modulation of mTORC1 signaling [196], while SLC7A5 and SLC7A8 are pre-primed in innate lymphoid cell type 2 (ILC2) populations and are indispensable for their expansion and cytokine production [197]. Glutamine competition further extends this nutrient axis to antigen-presenting and myeloid compartments. Tumor cells outcompete intratumoral myeloid cells for glutamine via SLC1A5, inducing ER stress and activating the IRE1α–XBP1 pathway. This stress-adaptive response promotes the accumulation of immunosuppressive GPR109A^+^ granulocytic myeloid-derived suppressor cells (G-MDSCs) and M2-like TAMs, thereby dampening CD8^+^ T cell–mediated antitumor immunity [198]. Similarly, type-1 conventional dendritic cells (cDC1s), which are essential for cytotoxic T cell priming, compete with tumor cells for glutamine uptake through SLC38A2. Glutamine deprivation selectively impairs cDC1 licensing by disrupting FLCN–TFEB–dependent activation programs, reinforcing immune tolerance within the TME [199, 200].

In addition to direct nutrient competition, tumors exploit immune-derived metabolites to induce immunosuppressive reprogramming through shared transporter-mediated mechanisms. SLC13A3 has been identified as a critical itaconate transporter that mediates metabolic crosstalk between TAMs and tumor cells. By importing macrophage-derived itaconate, tumor cells activate the NRF2–SLC7A11 antioxidant axis, thereby acquiring resistance to ferroptosis and escaping immune-mediated cell death [201]. Tryptophan metabolism further contributes to immune suppression through metabolite hijacking. Interferon-γ–induced kynurenine is transported via SLC7A8 and the proton-assisted amino acid transporter PAT4, altering immune cell signaling and function [202]. At the transcriptional level, XBP1 directly regulates SLC38A2 expression to sustain glutamine uptake and preserve T cell function under metabolic stress [203]. Tumors not only outcompete immune cells for essential nutrients but also actively reprogram immune metabolism through convergent transporter-dependent mechanisms, consolidating immune suppression across innate and adaptive compartments.

These competitive and hijacking mechanisms, mediated by distinct SLC transporters, collectively generate a nutrient-poor and metabolically altered TME that constrains immune cell metabolic programming and antitumor function.

#### SLC-Driven immunosuppressive reprogramming and cell fate decisions

In the immune context, GLUT1 negatively correlates with immune checkpoint molecules like PD-1 and CTLA4, while positively correlating with CD44 [204]. Glycolytic signaling can further drive immune checkpoint expression, as GJB2 enhances glycolysis and NF-κB activation to induce PD-L1 expression through the HIF-1α–GLUT1 axis [205]. In tamoxifen-resistant breast cancer cells, SGLT1 overexpression enhances glycolysis, increases lactic acid secretion, and promoting M2-like TAM polarization via the HIF-1α/STAT3 pathway [206]. Additionally, SLC3A2 facilitates arachidonic acid secretion in lung adenocarcinoma, contributing to TAM M2 polarization [207].

In HCC, SLC7A2 deficiency activates the PI3K/Akt/NF-kB pathway, leading to CXCL1 upregulation and recruitment of MDSCs, which foster tumor immunosuppression [208]. This mechanism is particularly relevant in virus-associated HCC, where SLC7A2 downregulation is frequently observed [209]. In osteosarcoma, chemotherapy-induced IL-18 secretion from macrophages upregulates LAT2 in tumor cells, enhancing leucine and glutamine uptake to activate mTORC1–c-Myc signaling and drive CD47 expression. This metabolic control of the “don’t eat me” signal suppresses macrophage phagocytosis and promotes immune escape [210].

In CRC, the dopamine transporter DAT (SLC6A3) sustains cancer stem cell programs by maintaining expression of the histone methyltransferase G9a. Genetic or pharmacological antagonism of DAT suppresses stemness, disrupts pluripotency-associated transcriptional networks, and enhances tumor immunogenicity through activation of endogenous retroelements and type I interferon signaling, thereby promoting lymphocytic infiltration [211].

T cell function and persistence are critically constrained by glucose availability and lactate accumulation. GLUT10 plays a selective role in CD8^+^ T cells, where lactic acid directly binds and inhibits GLUT10-mediated glucose transport, impairing effector function [212]. The low-affinity glucose transporter GLUT2 regulates CD8^+^ T cell effector differentiation by coupling glucose uptake, glycolysis, and intracellular glucose storage to environmental cues such as glucose availability, hypoxia, and extracellular acidification [213]. In the glucose-deprived TME, where tumor cells actively consume glucose, MCT1 facilitates lactic acid absorption in Tregs. This triggers NFAT1 nuclear translocation and enhances PD-1 expression in Tregs, while paradoxically suppressing PD-1 in effector T cells [214]. Treg cells exhibit metabolic flexibility by utilizing lactate as an alternative carbon source. Lactate uptake through the monocarboxylate transporter MCT1 sustains Tregs proliferation and suppressive function specifically within tumors. Genetic ablation of MCT1 in Tregs compromises intratumoral immune suppression, slows tumor growth, and enhances responsiveness to immunotherapy [215]. Notably, blocking MCT1 has been shown to enhance CAR T-cell therapy efficacy in treating B-cell malignancies [216]. MCT4 overexpression enhances lactic acid production and efflux, suppressing CD8^+^ T cell function and reducing the efficacy of anti-PD-1 inhibitors [217]. Therapeutically, targeting MCT4 shows promise. Inhibition of MCT4 reduces lactate efflux in CRC spheroids, enhancing ICB efficacy [218]. Notably, genetic ablation of MCT4 reverses PD-1 blockade resistance driven by LKB1/STK11 loss in syngeneic lung adenocarcinoma models [219].

Terminal T cell exhaustion is also metabolically enforced. MCT11 (SLC16A11) is selectively upregulated in terminally exhausted T cells, and genetic or antibody-mediated targeting of MCT11 reduces lactate uptake, restores effector function, and improves tumor control [220].

Extracellular acidosis within the TME impairs methionine uptake by downregulating SLC7A5, leading to altered histone modifications at promoters of T cell stemness-associated genes and reinforcing dysfunctional differentiation states [221]. In CD4^+^ T cells, SLC7A5 expression is regulated by SRC2 through c-Myc–dependent transcriptional programs [222], while the ATF4–SLC7A11-GSH axis contributes to immunosuppressive properties under arginine-depleted conditions [223]. Engineering CAR T cells by modulating SLC7A5 or SLC7A11 expression enhances therapeutic efficacy through altered arginase-dependent metabolic control [224].

Under conditions of tryptophan depletion, commonly observed in IDO1/TDO-expressing tumors, stress-responsive GCN2 signaling induces the amino acid transporter SLC7A5 (LAT1), thereby enhancing cellular uptake of kynurenine. Elevated kynurenine import sensitizes aryl hydrocarbon receptor (AHR) signaling, even in response to weak endogenous ligands, ultimately promoting regulatory T cell differentiation and suppressing antitumor immunity [225].

In multiple myeloma, excessive long-chain fatty acid uptake via the SLC-associated transporter FATP1 metabolically reprograms bone marrow CD8^+^ T cells, resulting in mitochondrial dysfunction and impaired antitumor activity. Targeting FATP1 rescues T cell metabolic fitness and function [226]. In tumor-infiltrating CD8^+^ T cells, CD36 facilitates fatty acid uptake, leading to lipid peroxidation and ferroptosis, which compromise cytotoxic function and antitumor capacity. Targeting CD36 or the ferroptosis pathway enhances CD8^+^ T cell antitumor responses and improves anti-PD-1 immunotherapy efficacy [227]. In contrast, CD36 is selectively upregulated in intratumoral Tregs, where it acts as a central metabolic regulator, optimizing mitochondrial fitness through PPAR-β signaling. This adaptation enables Tregs to thrive in the lactate-rich TME. Deletion of Cd36 in Tregs suppresses tumor growth by reducing Tregs infiltration and enhancing tumor-infiltrating lymphocyte (TIL) activity, while preserving systemic immune homeostasis [228].

Myeloid cell fate within the TME is extensively shaped by SLC-mediated metabolic inputs. Tumor-derived signals activate the PERK–ATF4 axis to induce GLUT1 expression in monocyte-derived macrophages, suppressing antitumor immunity in glioblastoma [229]. In contrast, GLUT3 promotes alternative macrophage polarization through a glucose transport–independent mechanism involving RAS-mediated endocytosis and IL-4–STAT6 signaling [230]. The cystine/glutamate antiporter xCT further governs macrophage fate; its deletion limits TAM recruitment, inhibits M2 polarization, and induces TAM ferroptosis, thereby suppressing tumorigenicity and metastasis in HCC models [231, 232].

Lipid transport pathways are equally influential. Single-cell transcriptomic analyses reveal selective upregulation of FABP1 in TAMs in advanced HCC, where it cooperates with the PPARG–CD36 axis to enhance fatty acid oxidation and enforce immunosuppressive polarization [233]. In liver metastasis, CD36-mediated uptake of tumor-derived lipid vesicles fuels macrophage lipid accumulation and M2-like polarization, correlating with immune exclusion and aggressive disease [234]. Neutrophils exhibit marked metabolic flexibility, sustaining survival and effector functions under nutrient deprivation through gluconeogenesis and glycogen cycling, providing a metabolic foundation upon which tumor-derived signals can impose immunosuppressive reprogramming [235, 236]. Tumor-associated neutrophils display increased GLUT1 expression and glycolytic activity. Neutrophil-specific deletion of GLUT1 reduces TAN accumulation, suppresses tumor growth, and enhances radiotherapy efficacy [237]. Tumor-derived lactate further drives accumulation of PD-L1^+^ neutrophils via MCT1-dependent activation of the NF-κB–COX-2 axis, dampening T cell cytotoxicity [238]. In CRC, region-specific CD16^+^ neutrophils acquire a pro-tumor phenotype through excessive cholesterol uptake mediated by scavenger receptors, including CD36 and LRP1, suppressing natural killer cells cytotoxic signaling and promoting immune evasion [239].

Dendritic cell function is likewise metabolically constrained. Coordinated expression of SLC7A5, SLC3A2, and SLC7A11 sustains mTORC1-driven anabolic and cytokine programs in plasmacytoid DCs [240]. Autophagy deficiency in DCs induces aberrant upregulation of CD36, leading to excessive lipid uptake that disrupts MHC class II antigen presentation and selectively impairs CD4^+^ T cell priming [241]. In lipid-rich pathological contexts, FATP2 can couple fatty acid uptake to inflammasome activation in MDSCs. Arachidonic acid transported via FATP2 triggers mitochondrial dysfunction and NLRP3 inflammasome activation, promoting IL-17–producing CD4^+^ T-cell responses and tumor recurrence following steatotic liver transplantation [242]. FATP2 is pathologically upregulated in polymorphonuclear myeloid-derived suppressor cells (PMN-MDSCs) and serves as a metabolic determinant of their immunosuppressive function. FATP2-driven uptake of arachidonic acid fuels prostaglandin E2 synthesis, thereby inhibiting T-cell responses and promoting tumor progression. Genetic or pharmacological inhibition of FATP2 abolishes PMN-MDSC suppressive activity and synergizes with ICB, identifying FATP2 as a selective immunometabolic target [243]. Tumor-derived exosomes further reinforce PMN-MDSC suppressive programs by upregulating FATP2. In bladder cancer, exosomal circRNA_0013936 enhances FATP2 expression while repressing RIPK3 in PMN-MDSCs via distinct miRNA–JAK2 and miRNA–CREB1 pathways. This dual regulation amplifies lipid uptake–driven immunosuppression and dampens CD8^+^ T-cell function [244].

#### SLC-Orchestrated stromal crosstalk and spatial metabolic niches

**Fig. 5:**
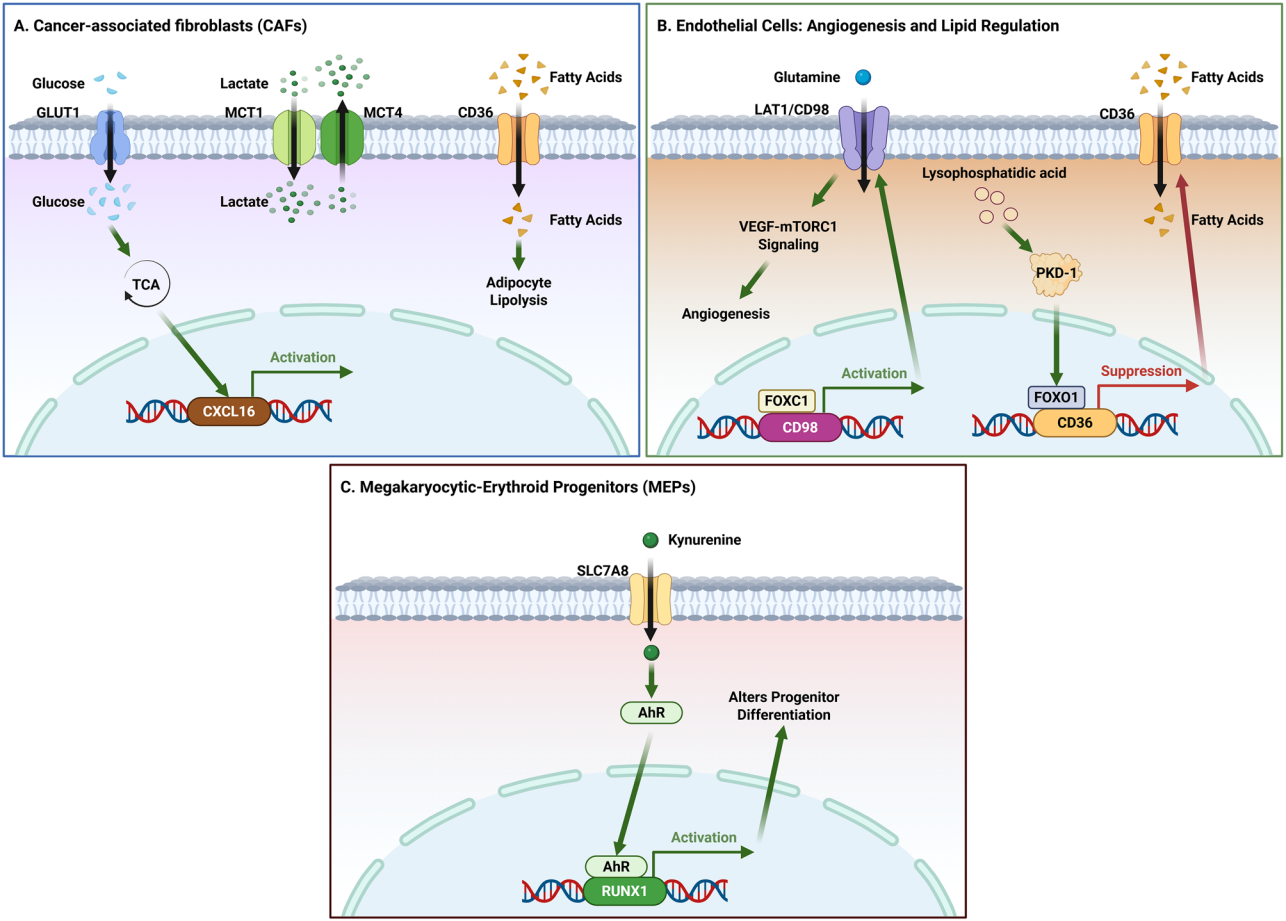
Functions of SLC transporters in stromal cells of the tumor microenvironment. (**A**) Cancer-associated fibroblasts (CAFs). GLUT1 activity is linked to CXCL16 expression. MCT1 and MCT4 mediate lactate exchange. CD36 promotes fatty acid uptake and adipocyte lipolysis. (**B**) Endothelial cells. FOXC1 transcriptionally upregulates the LAT1/CD98 heterodimer (SLC7A5/SLC3A2), enhancing VEGF-mTORC1 signaling for angiogenesis. LPA signaling via PKD-1 and FOXO1 represses CD36 expression. (**C**) Megakaryocytic-erythroid progenitors (MEPs). SLC7A8-mediated kynurenine import activates the AhR-RUNX1 transcriptional axis to regulate differentiation

Beyond tumor-intrinsic metabolic rewiring, SLC transporters play a central role in coordinating metabolic crosstalk between cancer cells and diverse stromal populations, thereby establishing spatially organized metabolic niches that support tumor growth, immune evasion, and metastatic dissemination (Fig. 5). Among stromal compartments, CAFs represent a primary interface through which transporter-dependent metabolic programs are propagated within the TME. In NSCLC, CAF-derived exosomal METTL3 induces m6A-dependent stabilization of SLC7A5 in tumor cells, reinforcing glutamine uptake and glutaminolysis to sustain tumor growth and stemness [245]. CAF metabolic states also influence immune cell exclusion. Glycolytic CAF (GlyCAF) subtypes employ GLUT1-dependent CXCL16 expression to impede cytotoxic T cell infiltration into the tumor parenchyma [246]. Conversely, genetic deletion of GLUT1 in CAFs suppresses the expression of tumor-promoting factors and reshapes tumor cell transcriptional programs, resulting in reduced liver metastasis in CRC models [247]. CAFs also contribute to metabolic changes by exporting lactate through MCT4, which reshapes the metabolic landscape of both stromal and epithelial compartments within the TME [248]. These observations highlight SLC transporters as metabolic interfaces that translate CAF metabolic states into coordinated changes in tumor cell nutrient acquisition, signaling, and immune accessibility.

Stromal metabolic crosstalk is further spatially refined in adipocyte-rich microenvironments, where lipid availability defines specialized metabolic niches. Stromal lipid release induces CD36 expression in breast cancer cells, facilitating fatty acid uptake from neighboring adipocytes. CD36 directly interacts with FABP4 to coordinate lipid import and intracellular trafficking, which promotes fatty acid oxidation, epithelial–mesenchymal transition, and stemness [249]. Consistent with this spatial lipid transfer model, single-cell transcriptomic analyses in CRC identify a distinct subpopulation of CD36^+^ CAFs [250]. In these cells, adipocyte lipolysis triggers a cascade involving CD36 modifications, including deacylation, deglycosylation, and dissociation from interacting proteins such as prohibitin-1 (PHB) and annexin 2 (ANX2), further illustrating the dynamic metabolic reprogramming within the TME [251]. These findings exemplify how SLC-dependent lipid transfer establishes spatially restricted metabolic niches that couple local nutrient availability to tumor cell plasticity and invasive potential.

The tumor vasculature further influences the metabolic landscape. Endothelial expression of the amino acid transporter LAT1 promotes angiogenesis by regulating endothelial proliferation and enhancing VEGF-A–dependent activation of mTORC1 [252]. Transcriptional regulation further refines endothelial metabolic states. FOXC1 controls expression of the LAT1 complex during angiogenesis and blood–retina barrier formation [253]. The LPA/PKD-1-FoxO1 signaling axis also mediates endothelial CD36 repression, which contributes to proangiogenic reprogramming in the tumor vasculature [254]. Together with CAF- and adipocyte-associated programs, endothelial SLC regulation reinforces the concept that diverse stromal compartments converge on shared transporter-mediated mechanisms to shape spatial metabolic organization within tumors.

Beyond local stromal interactions, SLC-dependent metabolic crosstalk can extend to circulating and progenitor cell compartments, shaping pro-metastatic niches. In HCC, tumor-derived periostin (POSTN) promotes mobilization and tumor infiltration of endothelial progenitor cells (EPCs). Through an αvβ3–ILK–NF-κB axis, POSTN induces EPC-derived CCL2 secretion, which in turn activates CCR2–STAT3 signaling in tumor cells to transcriptionally upregulate the fatty acid transporter CD36 [255]. This mechanism illustrates how SLC-mediated metabolic communication extends beyond the local stroma to coordinate systemic and pre-metastatic niche formation. Metabolic crosstalk mediated by SLC transporters also extends beyond the immediate tumor stroma to influence systemic progenitor compartments. Tumor-derived kynurenine is imported into megakaryocytic–erythroid progenitors via SLC7A8, activating an AhR–RUNX1 transcriptional program that alters progenitor differentiation in both humanized mouse models and cancer patients [256]. In contrast, selective ablation of SLC7A11 in tumor cells alone fails to impair tumor growth in transgenic PDAC models, underscoring the dominant contribution of stromal and microenvironmental metabolic inputs to tumor responses under metabolic stress [257].

### Therapeutic applications and novel strategies

#### Metabolic adaptation–driven resistance

Targeting glucose transport represents a prototypical approach to counteract glycolysis-dependent resistance. Genetic silencing of GLUT1 synergizes with curcumin to enhance radiosensitivity by simultaneously inducing apoptosis and autophagy [258]. Consistently, the small-molecule GLUT1 inhibitor BAY-876 effectively reduces glucose uptake, promotes apoptotic cell death, and enhances sensitivity to cisplatin and other anticancer therapies [259, 260]. Notably, co-inhibition of GLUT1 synergizes with cytotoxic agents such as docetaxel by amplifying DNA damage and attenuating Akt–mTOR and MAPK signaling [261–263]. Another GLUT1 inhibitor, WZB117, has also demonstrated antitumor activity by inducing apoptosis in imatinib-resistant cancer cells [264]. Beyond single-transporter targeting, redundant glucose uptake pathways contribute to resistance robustness. Dual inhibition of GLUT1 and GLUT3 using the indomorphane derivative Glupin suppresses glucose-driven survival mechanisms more effectively than selective blockade [265].

SGLT2 inhibitors, such as canagliflozin, target resistance-associated metabolic adaptations through regulation of AMPK and mTOR signaling [266]. In prostate and lung cancer models, canagliflozin reduces glucose uptake, suppresses mitochondrial respiration, and inhibits cellular proliferation [267]. Similarly, empagliflozin exerts antitumor effects by modulating multiple oncogenic pathways, including PGC-1α, FOXO3a, and Sonic Hedgehog (SHH), thereby inhibiting cancer cell migration and promoting apoptosis [268, 269].

Resistance to therapy is tightly coupled to redox homeostasis. In GC, upregulation of ATF3 alleviates cisplatin resistance by promoting ferroptosis through suppression of the Nrf2–Keap1–xCT axis [270], while butyrate reverses ferroptosis resistance in CRC by suppressing xCT via c-Fos–dependent mechanism [271].

#### Transporter-controlled Drug bioavailability

Beyond metabolic adaptations, the therapeutic efficacy of antineoplastic agents is fundamentally governed by their intracellular bioavailability, a parameter stringently controlled by SLC-mediated drug influx [272]. Nucleoside transporters provide clinically actionable evidence for this paradigm: expression levels of equilibrative and concentrative nucleoside transporters, including human equilibrative nucleoside transporter 1 (hENT1) and human concentrative nucleoside transporter 3 (hCNT3), robustly predict treatment response in resected PDAC and chronic lymphocytic leukemia [273–275]. Reduced expression of hENT1 is a validated predictor of gemcitabine resistance in PDAC, driven by integrin signaling, epithelial–mesenchymal transition, and hypoxia-dependent transcriptional repression [276–278]. In CRC, upregulation of histone deacetylase 7 (HDAC7) induces hypoacetylation at the CNT2 (SLC28A2) promoter. Pharmacological inhibition of HDAC activity restores CNT2 expression, enhances nucleoside uptake, and sensitizes tumor cells to nucleoside analog chemotherapy [279].

Extending beyond nucleosides, members of the SLC22 and SLCO families are critical determinants of responsiveness to TKIs and platinum-based chemotherapy [280]. Traditionally, organic cation transporter 1 (OCT1, SLC22A1) has been recognized as a major influx pathway for TKIs, most notably imatinib and sorafenib [281, 282]. However, subsequent studies revealed OCT1-independent transport in HCC, challenging its utility as a predictive biomarker [281, 283]. Downregulation of organic cation transporters represents a recurrent mechanism of impaired drug influx. For instance, reduced expression of OCT6 (SLC22A16) has been linked to cisplatin resistance in lung cancer [284]. More broadly, several SLCs with tumor-suppressive functions are frequently silenced through promoter hypermethylation, including SLC5A8 in cervical cancer and OCT3 (SLC22A3) in AML, where epigenetic repression correlates with poor prognosis and therapeutic resistance [285, 286]. Pharmacological demethylation restores OCT3 expression and significantly enhances oxaliplatin efficacy in CRC [287]. In contrast, OCT2 (SLC22A2) mediates oxaliplatin uptake in dorsal root ganglia, linking transporter activity to dose-limiting neurotoxicity rather than tumor response, and competing for OCT2-mediated uptake using the TKI dasatinib may mitigate oxaliplatin-induced neurotoxicity [288]. Complementarily, several flavonoids have been identified as OCT2 inhibitors capable of attenuating cisplatin-induced cytotoxicity, providing a potential strategy to alleviate treatment-associated nephrotoxicity [289, 290].

Organic anion transporting polypeptides (OATPs), particularly OATP1B1 and OATP1B3, further modulate therapeutic response by governing the hepatic uptake and systemic clearance of multiple anticancer agents. These transporters are essential for the elimination of glucuronide conjugates, including sorafenib–glucuronide, through the process of “hepatocyte hopping”, a unique recycling mechanism that shapes in vivo drug redistribution and exposure [291, 292]. In vivo studies using Oatp1a/1b-deficient and liver-specific humanized mouse models demonstrate that OATP1B1, OATP1B3, and OATP1A2 directly mediate docetaxel clearance. Loss of OATP function results in markedly increased systemic exposure and exacerbated toxicity [292, 293]. These findings establish transporter-controlled drug bioavailability as a central determinant of therapeutic index and interpatient variability.

#### SLC transporters in cancer therapy related toxicities

**Fig. 6:**
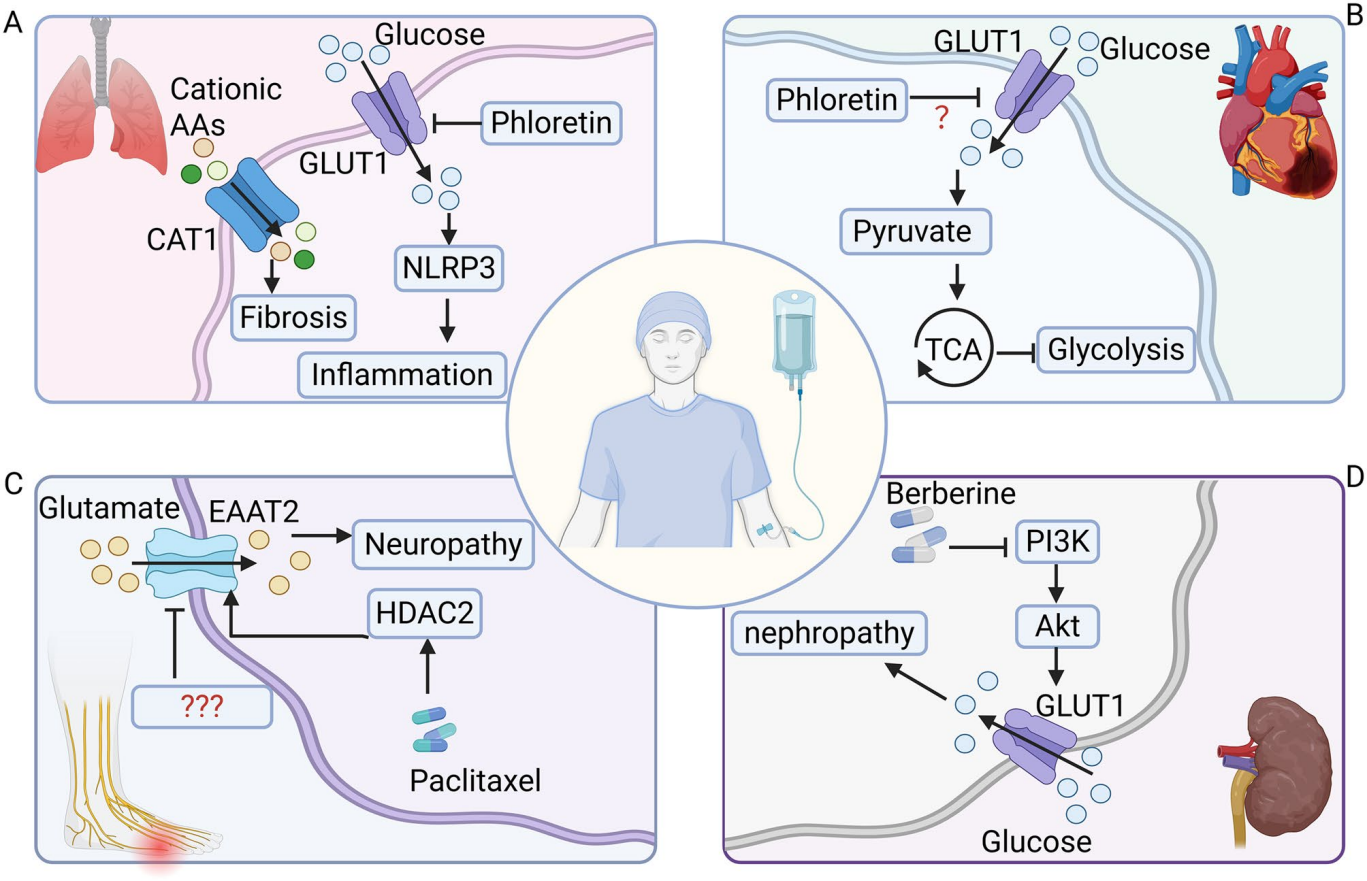
Slcs in therapy-related toxicities. (**A**) Pulmonary toxicity. GLUT1-mediated glucose uptake is associated with NLRP3 inflammasome activation and inflammation. The cationic amino acid transporter SLC7A1 (CAT1) is linked to fibrosis. The GLUT1 inhibitor phloretin is indicated. (**B**) Cardiac toxicity. GLUT1 facilitates glycolytic flux, contributing to hypertrophy. Its expression is regulated by the PI3K/Akt pathway. The natural compound berberine can target GLUT1. (**C**) Neurological toxicity. Paclitaxel treatment is associated with dysregulation of the glutamate transporter SLC1A2 (EAAT2), involving HDAC2, leading to neuropathy. (**D**) Nephrotoxicity. GLUT1 regulation occurs through the PI3K/Akt signaling axis, affecting mesangial cell proliferation. Berberine demonstrates therapeutic potential by modulating this pathway

While SLC transporters are frequently leveraged as therapeutic vulnerabilities in cancer, their indispensable physiological roles in normal tissues impose intrinsic constraints on therapeutic selectivity. Figure 6 provides an overview of major organ-specific toxicities associated with dysregulated SLC activity, including pulmonary, cardiac, neurological, and renal complications. In pulmonary tissues, GLUT1 contributes to acute lung injury by disrupting epithelial tight junction integrity and promoting NLRP3 inflammasome activation in airway epithelium [294, 295]. Pharmacological suppression of GLUT1-driven glycolysis using phloretin alleviates lung injury by dampening macrophage-mediated inflammatory responses [296]. Additionally, dihydromyricetin shows promise in alleviating pulmonary fibrosis through regulation of the STAT3/p-STAT3/GLUT1 signaling pathway [297]. In parallel, the CAT2 has been identified as a key regulator of inflammatory homeostasis [298], and fibrosis [299] in the lung.

Cardiovascular toxicity represents another major off-tumor liability associated with dysregulated glucose transport. GLUT1 has been implicated in cardiac hypertrophy during acute pressure overload, where excessive glycolytic flux leads to accumulation of metabolic intermediates and maladaptive remodeling [300]. Dysregulation of the GLUT1–PKM2 metabolic loop promotes metainflammation in patients with non–ST-segment elevation myocardial infarction [301], while ARRDC4–GLUT1 interactions mediate metabolic stress responses in ischemic myocardium [302].

Renal and neurological toxicities further illustrate the systemic consequences of transporter perturbation. Berberine attenuates diabetic nephropathy by regulating mesangial cell proliferation through the PI3K/Akt/AS160/GLUT1 signaling pathway [303]. In the nervous system, HDAC2-mediated repression of EAAT2 has been linked to paclitaxel-induced painful neuropathy [304].

Beyond organ-specific toxicities, accumulating evidence indicates that off-tumor SLC biology can be therapeutically exploitable. Notably, SGLT2 inhibitors exhibit a unique dual benefit in cardio-oncology patients, providing both cardioprotective effects and anticancer activity [305]. Large-scale safety monitoring studies provide a nuanced perspective on transporter targeting. Multisite cohort analyses indicate that SGLT2 inhibitor use does not increase short-term bladder cancer risk and is associated with reduced prostate cancer incidence [306, 307]. In cardio-oncology settings, SGLT2 inhibitors improve outcomes in patients with cancer therapy–related cardiac dysfunction and reduce cardiac events in anthracycline-treated populations [308, 309]. BLF501, a synthetic compound with high SGLT-1 affinity, demonstrates protective effects against mucosal injuries in mice treated with DXR and/or 5-FU [310].

Emerging data further expand the relevance of SLCs beyond oncology. The L-type amino acid transporter LAT1 has been implicated in inflammatory and autoimmune conditions, where its inhibition in innate and adaptive T cells effectively controls skin inflammation, and ameliorates severe autoimmune arthritis in the SKG mouse model [311, 312]. These findings extend our understanding beyond cancer applications to broader therapeutic possibilities.

Additional mechanisms of toxicity and resistance continue to emerge. Suppression of Skp2 contributes to sepsis-induced acute lung injury by enhancing ferroptosis through SLC3A2 ubiquitination [313]. Myeloid FTH1 deficiency protects mice from colitis and colitis-associated CRC by reducing DMT1-imported iron and STAT3 activation under inflammation conditions [314]. Moreover, modulation of SLC7A11 improves dendritic cell–mediated efferocytosis and accelerates wound healing in diabetic settings [315, 316].

#### Combinatorial therapeutic paradigms

##### SLC-Dependent metabolic liabilities and therapeutic sensitization

SLC-centered strategies also exploit intrinsic metabolic liabilities that shape tumor sensitivity to targeted agents and cytotoxic chemotherapy. Disruption of glucose–lactate flux represents a central strategy for amplifying metabolic stress. Broad inhibition of facilitative glucose transport by RgA suppresses glucose uptake, ATP production, and mTOR signaling, inducing cell cycle arrest and apoptosis [317]. MCT1 inhibition with AZD3965 has progressed to early-phase clinical evaluation in lymphoma, underscoring the translational feasibility of targeting glycolytic flux [318, 319]. Complementing lactate influx inhibition, blockade of lactate export via MCT4 further enhances therapeutic vulnerability. Combined targeting of MCT4 with metformin or NF-κB inhibitors significantly improves therapeutic efficacy in diabetes-associated breast cancer [320].

Beyond carbon flux, tumors exhibit pronounced dependencies on nitrogen acquisition and mitochondrial metabolite exchange that can also be therapeutically exploited. Inhibition of the mitochondrial citrate transporter SLC25A1 by parthenolide selectively suppresses liver cancer stem cell growth [321]. Amino acid transport–driven anabolic programs further shape therapeutic sensitivity. In CRC, metformin suppresses MYC-dependent expression of the large neutral amino acid transporter SLC7A5, thereby limiting tryptophan uptake and reinforcing metabolic stress under treatment [322]. Similarly, NDRG2- mediated repression of SLC1A5, which inhibits glutaminolysis, has emerged as a promising target to block metastatic tumor survival [323]. These nitrogen-dependent vulnerabilities can also be leveraged for immunotherapeutic targeting. SLC3A2-specific CAR T cells significantly improve survival in prostate cancer models by exploiting transporter overexpression as a selective metabolic and antigenic vulnerability [324].

In addition to facilitative glucose transport, sodium-dependent glucose uptake has emerged as a distinct metabolic entry point that can be therapeutically exploited, particularly under glucose-limited conditions. In pancreatic cancer xenograft models, SGLT2 inhibitors effectively block glucose uptake [325]. Consistent with these preclinical observations, analysis of a large SEER–Medicare–linked cohort revealed improved overall survival among patients with NSCLC and pre-existing diabetes who received SGLT2 inhibitors [326]. Moreover, selective targeting of SGLT2 using gliflozins has been shown to significantly reduce tumor growth and prolong survival in both autochthonous mouse models and patient-derived xenografts (PDX) of lung adenocarcinoma (LUAD) [327]. 

##### Combination with ICB

Transporter-targeted metabolic interventions also provide a compelling strategy to enhance immune checkpoint blockade (ICB) efficacy. Alleviation of nutrient competition represents a major mode of synergy. Pharmacological inhibition of GLUT1 using BAY-876 synergizes with PD-1/PD-L1 blockade in orthotopic glioblastoma models to delay tumor growth and prolong survival by reducing tumor glycolysis, diminishing lactate accumulation, and restoring immune responsiveness [328]. Similarly, inhibition of the glutamine transporter SLC1A5 enhances therapeutic sensitivity in multiple myeloma and CRC, where elevated SLC1A5 expression is associated with treatment resistance. Targeting SLC1A5 potentiates cetuximab efficacy in CRC, supporting amino acid transport as a metabolic vulnerability that can be exploited to improve antitumor responses [86, 329].

Beyond nutrient availability, certain SLC transporters directly modulate immune checkpoint stability. Canagliflozin disrupts the SGLT2–PD-L1 interaction, promoting PD-L1 degradation and functionally enhancing antitumor immunity in preclinical models [330]. In pancreatic cancer, inhibition of the bicarbonate transporter SLC4A4 alleviates tumor-associated acidosis, restores T-cell activity, and improves responsiveness to ICB [331]. Metabolic reprogramming of immunosuppressive myeloid populations represents another mechanism of synergy. Inhibition of the lipid transporter FATP2 reduces myeloid-derived suppressor cell–mediated immunosuppression and significantly enhances anti-PD-L1 efficacy by restoring CD8^+^ T-cell function [332].

Notably, combinations involving the cystine–glutamate antiporter SLC7A11 exhibit pronounced context dependency. In breast cancer and hepatocellular carcinoma, SLC7A11 inhibition enhances immunotherapy responses, partly through ferroptosis-associated remodeling of the tumor immune microenvironment [231, 333]. In contrast, in melanoma, SLC7A11 inhibition paradoxically attenuates anti-PD-1 efficacy by promoting PD-L1–enriched exosome release and macrophage M2 polarization [334]. Restoration of redox balance through enforced expression of glutamate–cysteine ligase catalytic subunit (GCLC) promotes glutathione synthesis, counteracts ferroptosis, prevents CD36 upregulation, and ultimately enhances T-cell–mediated antitumor responses, underscoring the nuanced interplay between redox metabolism, lipid uptake, and immune regulation [335].

##### Combination with radiotherapy

Radiotherapy imposes profound metabolic stress on tumor cells, creating additional opportunities for transporter-targeted therapeutic synergy. Transporter-based combinations with radiotherapy primarily exploit two interconnected vulnerabilities: amplification of oxidative and ferroptotic stress, and disruption of hypoxia-driven metabolic adaptation. Inhibition of the cystine–glutamate antiporter SLC7A11 amplifies radiation-induced ferroptosis and lipid peroxidation, sensitizing tumors to irradiation [336, 337]. Radiotherapy-induced metabolic rewiring can further expose ferroptosis-associated liabilities. Studies in HNSCC have revealed that radiation increases glutamine levels and upregulates the glutamine transporter SLC1A5. Combining glutamine inhibition with radiation induces immunogenic tumor ferroptosis [338]. Clinical observations further support the relevance of amino acid transport in radiotherapy outcomes, as low expression of SLC3A2 and SLC7A5/LAT1 correlates with improved prognosis following radiochemotherapy in HNSCC [339].

In parallel, transporter-targeted strategies can disrupt hypoxia-driven metabolic adaptation and glycolytic resilience following irradiation. In small cell lung cancer, combining the MCT1 inhibitor AZD3965 with fractionated radiotherapy significantly improves therapeutic efficacy compared with either modality alone [340]. Synergistic interactions have also been observed between SLC7A11 inhibition and PI3K/Akt pathway targeting in combination with DNA-damaging agents such as Riluzole–Pt(IV) prodrugs [341]. Consistent with the importance of hypoxia-adaptive glucose utilization, SGLT2 inhibitors, including canagliflozin, enhance radiotherapy responses by suppressing HIF-1α signaling in prostate cancer and non–small cell lung cancer [342, 343]. To further improve therapeutic delivery, canagliflozin-loaded magnetic nanoparticles have been developed to target hypoxic tumors in combination with radiotherapy [344].

#### Critical appraisal of clinical trials and translational barriers

Although several SLC-directed agents have entered early-phase clinical evaluation (Table 1), most studies remain focused on safety and feasibility rather than definitive efficacy. A fundamental limitation of current trial designs lies in the recruitment of heterogeneous patient populations defined primarily by tumor histology, often without stratification by metabolic phenotype or baseline expression of the targeted transporter. Consequently, a substantial proportion of enrolled patients may harbor tumors that are not biologically dependent on the targeted SLC, thereby diluting potential therapeutic signals—a challenge particularly pronounced in Phase I studies with limited statistical power and slow accrual.

**Table 1:** Summary of clinical trials targeting SLC transporters in cancer therapy

Clinical trials.gov identifier	Title	Drug	Target	Histological type of cancer	study type	Phase	Interventions	Results
NCT04542291	Targeting Pancreatic Cancer With Sodium Glucose Transporter 2 (SGLT2) Inhibition	dapagliflozin	SGLT2	Pancreatic Cancer	Observational	1	Drug: Dapagliflozin Device: BIOSENSE meters	Well-tolerated. *N* = 12: 2 PR, 9 SD [345]
NCT04887935	Neoadjuvant SGLT2 Inhibition in High-Risk Localized Prostate Cancer	Dapagliflozin	SGLT2	Prostate Cancer	Interventional	1	Drug: Dapagliflozin	Not yet completed
NCT06341842	Potential Protective Role of SGLT-2 Inhibitors for Chemotherapy-induced Cardiotoxicity (PROTECT)	Dapagliflozin	SGLT2	Breast Cancer	Interventional	2	Drug: Dapagliflozin	Not yet completed
NCT05521984	Targeting Pediatric Brain Tumors and Relapsed/Refractory Solid Tumors With Sodium Glucose Cotransporter 2 Inhibitors (SGLT2i)	Dapagliflozin	SGLT2	Pediatric Brain Tumor	Interventional	1	Drug: Dapagliflozin Commercially available Drug: Carmustine Standard of care Drug: Topotecan Standard of care Drug: Cyclophosphamide Standard of care	No Results Posted
NCT06103279	Cardioprotective Empagliflozin for Cancer Patients Receiving Doxorubicin	Empagliflozin	SGLT2	Cancer	Interventional	2/3	Drug: Empagliflozin	Not yet recruiting
NCT04073680	A Phase 1b/2 Study of Serabelisib in Combination With Canagliflozin in Patients With Advanced Solid Tumors	Canagliflozin	SGLT2	Advanced Solid Tumor	Interventional	1/2	canagliflozin in combination with serabelisib	Not yet completed
NCT06711185	Effect of DAPAglifozin on MYOcardial Remodeling of Breast CANCER Patients Treated with Anthracycline Based Chemotherapy (DAPA-MYOCANCER)	Dapagliflozin	SGLT2	Breast Cancer	Interventional	3	Drug: Dapagliflozin Other: Placebo	Not yet completed
NCT04481256	Canagliflozin With Gemcitabine in Pancreatic Carcinoma	Canagliflozin	SGLT2	Pancreatic Carcinoma	Interventional	Not Applicable	Drug: Canagliflozin and Gemcitabine Drug: Gemcitabine	Not yet completed
NCT05025735	Alpelisib, Fulvestrant and Dapagliflozin for the Treatment of HR+, HER2 -, PIK3CA Mutant Metastatic Breast Cancer	Dapagliflozin	SGLT2	Metastatic Breast CancerHER2-negative Breast Cancer	Interventional	2	Drug: Dapagliflozin	Not yet completed
NCT04899349	Study of Safety and Efficacy of Dapagliflozin + Metformin XR Versus Metformin XR in Participants With HR+, HER2-, Advanced Breast Cancer While on Treatment With Alpelisib and Fulvestrant (EPIK-B4)	Dapagliflozin	SGLT2	Advanced Breast Cancer	Interventional	2	Experimental: Alpelisib + Fulvestrant + Dapagliflozin + Metformin XR Active Comparator: Alpelisib + Fulvestrant + Metformin XR	Study was early terminated due to slow recruitment and emerging data showing that prophylactic use of metformin may prevent or reduce the incidence of all-grades alpelisib-related hyperglycemia. The decision was not driven by safety concerns
NCT06304857	CardioPROTECTion with Dapagliflozin in Breast Cancer Patients Treated with AnthrAcycline - PROTECTAA TRIAL (PROTECTAA)	Dapagliflozin	SGLT2	Breast Cancer	Interventional	3	Drug: Dapagliflozin Drug: Placebo	Not yet completed
NCT06427226	Evaluation of the Possible Safety and Efficacy of Dapagliflozin in the Prophylaxis of Doxorubicin-Induced Cardiotoxicity	Dapagliflozin	SGLT2	Breast Cancer	Interventional	2	Drug: Dapagliflozin	Not yet recruiting
NCT05271162	Empagliflozin in the Prevention of Cardiotoxicity in Cancer Patients Undergoing Chemotherapy Based on Anthracyclines (EMPACT)	Empagliflozin	SGLT2	Cancer	Interventional	3	Drug: Empagliflozin	Not yet completed
NCT01791595	A Phase I Trial of AZD3965 in Patients With Advanced Cancer	AZD3965	MCT1	Adult Solid Tumor Diffuse Large B Cell Lymphoma Burkitt Lymphoma	Interventional	1	Drug: AZD3965	Well tolerated, Grade 3 cardiac troponin rise and acidosis observed at ≥20 mg/day [346].
NCT04430842	Dose Escalation Study to Assess the Safety, Tolerability, Pharmacokinetics, and Pharmacodynamics of QBS10072S	QBS10072S	LAT1	Advanced or Metastatic Cancers	Interventional	1	Drug: QBS10072S	None
UMIN000034080		Nanvuranlat (JPH203)	LAT1	Pretreated Advanced Refractory Biliary Tract Cancer	Interventional	2	Drug: Nanvuranlat	Nanvuranlat vs Placebo PFS: HR, 0.56 (95% CI, 0.34–0.90), *p* = 0.02; G ≥ 3 AEs : 30% vs 22.9% [347]
NCT02040506	A Phase I Study of IGN523 in Subjects With Relapsed or Refractory AML	IGN523	SLC3A2	Acute Myelogenous Leukemia AML	Interventional	Phase 1	Drug: IGN523	None
NCT05580861	Sulfasalazine in AML Treated by Intensive Chemotherapy: Elderly Patients-first Line Treatment (SALMA)	sulfasalazine	xCT	AML	Interventional	Phase 1 Phase 2	Drug: Sulfasalazine	Not yet completed
NCT01198145	Sulfasalazine in Preventing Acute Diarrhea in Patients With Cancer Who Are Undergoing Pelvic Radiation Therapy	Sulfasalazine	xCT	Patients With Cancer Who Are Undergoing Pelvic Radiation Therapy	Interventional	Phase 3	Drug: sulfasalazine	Sulfasalazine does not reduce enteritis during pelvic RT and may be associated with a higher risk of adverse events than placebo.
NCT04205357	Sulfasalazine and Stereotactic Radiosurgery for Recurrent Glioblastoma (SAS-GKRS)	Sulfasalazine	xCT	Recurrent Glioblastoma	Interventional	phase 1	Drug: Sulfasalazine	sulfasalazine sensitizes gliomas to gamma knife radiosurgery
NCT03847311	Sulfasalazine in Decreasing Opioids Requirements in Breast Cancer Patients	Sulfasalazine	xCT	Breast Cancer	Interventional	phase 2	Drug: Sulfasalazine	None
NCT06134388	Sulfasalazine in Patients With Metastatic CRC	Sulfasalazine	xCT	Metastatic CRC	Interventional	Phase 3	Drug: Sulfasalazine	Not yet completed
NCT01577966	Pilot Study Effect of Sulfasalazine on Glutamate Levels by (Magnetic Resonance Spectroscopy) MRS in Patients With Glioma	Sulfasalazine	xCT	Brain Tumor	Interventional	pilot study	Drug: Sulfasalazine	Completed

Importantly, several trials were terminated not due to unacceptable toxicity or a lack of biological rationale, but because of practical translational barriers such as slow patient recruitment and shifting standards of care. For instance, the early termination of studies involving SGLT2 inhibitors (e.g., dapagliflozin) was driven largely by accrual challenges and emerging evidence that alternative metabolic management could mitigate treatment-related hyperglycemia, rather than intrinsic safety concerns. These outcomes underscore the inherent difficulty of demonstrating incremental benefits for SLC-targeted interventions in unstratified clinical settings where functional redundancy within the SLC family may allow tumor cells to bypass single-transporter inhibition.

Notably, select studies—such as those targeting LAT1 in advanced biliary tract cancer—have demonstrated preliminary efficacy signals, reinforcing the concept that SLCs represent actionable vulnerabilities when biological dependency and trial design are appropriately aligned. Collectively, the inconsistent clinical performance of SLC-targeted therapies to date does not necessarily reflect inadequate biological relevance, but rather underscores a need for next-generation trial designs. Future strategies must integrate transporter expression profiling, functional pharmacodynamic readouts, and rational biomarker-guided patient selection to fully realize the therapeutic potential of SLC-centered precision oncology.

#### Next-generation strategies for SLC targeting

Despite strong biological rationale, functional redundancy, broad tissue distribution, and compensatory regulation of SLCs pose major translational challenges for therapeutic targeting [348, 349]. These constraints necessitate next-generation strategies that prioritize precision, durability, and spatial selectivity [350, 351]. Importantly, emerging systems-level analyses indicate that SLC dependencies are not static but can be dynamically reshaped under therapeutic pressure. Recent advances in computational biology, including spatiotemporal modeling of drug resistance, have identified treatment-emergent dependencies on SLC38A7 and SLC46A1 during breast cancer evolution [352].

Targeted protein degradation represents a powerful alternative to reversible inhibition in this context [353, 354]. PROTACs function as heterobifunctional degraders that bridge a protein of interest and an E3 ubiquitin ligase via a specialized linker, inducing proximity-dependent ubiquitylation and proteasomal elimination of the target protein, while enabling catalytic reuse of the degrader molecule [355]. Using a dTAG-based platform, selective degradation of multiple SLC family members disrupts intracellular pH homeostasis and suppresses tumor growth in vitro [356, 357].

Notably, a peptide-based PROTAC targeting the transcription factor FOXM1 induces efficient FOXM1 degradation while concomitantly downregulating GLUT1 and PD-L1, thereby impairing glucose metabolism and immune evasion without detectable toxicity in normal tissues [358]. This example highlights how indirect targeting of SLC-driven programs may achieve durable metabolic and immunological effects.

Novel therapeutic approaches targeting SLC7A11 have shown promising results in cancer treatment. For instance, the compound HG106 selectively induces cytotoxicity in KRAS-mutant cells by triggering oxidative stress and ER stress-mediated apoptosis. This mechanism leads to significant tumor suppression and prolonged survival in preclinical lung adenocarcinoma models [359]. In addition to these effects, targeting the SLC7A11/glutathione axis has been shown to induce ferroptosis and apoptosis in nasopharyngeal carcinoma. This approach not only disrupts cellular redox balance but also alters MAPK signaling [360].

Transporter-hijacking strategies further repurpose SLCs as active delivery portals. GLUT-targeting chimeras (GTACs) exploit glucose transporter–mediated uptake to induce lysosomal degradation of extracellular targets such as TNF-α and HER2 [361]. Complementary to degradation-based strategies, nanoparticle-mediated delivery systems offer a powerful means to address both redundancy and off-tumor toxicity by exploiting tumor-specific SLC expression patterns. LAT1-targeted liposomes loaded with chemotherapeutic agents such as paclitaxel or methotrexate achieve preferential tumor accumulation and reduced systemic toxicity in preclinical models [362]. Similarly, OATP1B3-functionalized nanoparticles enhance docetaxel delivery in HCC by leveraging the aberrant overexpression of SLCO1B3 in liver tumors [363].

Differential expression of SLCs in normal versus malignant tissues has enabled transporter-guided drug delivery strategies based on substrate mimicry. Bile acid transporters of the SLC10 family, including NTCP (SLC10A1) and ASBT (SLC10A2), facilitate uptake of bile acid–conjugated chemotherapeutics such as bendamustine and irinotecan [364, 365]. Gemcitabine-loaded glycocholic acid–modified micelles exploit ASBT-mediated transport to achieve oral bioavailability exceeding 81% and superior antitumor efficacy compared with free drug [366, 367]. Expanding beyond the gastrointestinal tract, LAT1 serves as a critical portal for broad-spectrum tumor targeting and blood–brain barrier penetration [368, 369]. LAT1 is highly expressed in the cerebral cortex, blood–brain barrier, placenta, bone marrow, and a wide range of malignancies. Clinically used agents, including L-DOPA, melphalan, and gabapentin, exploit LAT1-mediated transport to reach their target tissues, providing proof-of-concept for LAT1-guided drug delivery strategies [370]. Recent innovations include water-soluble large amino acid mimicking carbon quantum dots (LAAM GSH-CQDs) for tumor-specific targeting and carrier-free pro-protein platforms for cancer-selective cytosolic delivery via reversible tagging with LAT1 substrates [371, 372].

Crucially, the success of next-generation SLC-targeting strategies will depend on the integration of companion diagnostics and patient stratification. Given the heterogeneity of SLC expression across tumor types and disease stages, biomarker-guided selection of patients most likely to benefit from specific transporter-directed interventions is essential. Transcriptomic, proteomic, and functional imaging approaches capable of assessing transporter activity in vivo will play an increasingly important role in guiding therapeutic decisions and minimizing unintended toxicity.

## Discussion

The therapeutic targeting of SLC transporters has transitioned from a compelling metabolic hypothesis to a frontier of intense preclinical and clinical investigation. While the dysregulation of SLCs is a ubiquitous hallmark of cancer, the journey from genetic association to effective therapy is fraught with context-specific challenges. By synthesizing lessons from prototypic malignancies and dissecting the multidimensional roles of SLCs in the TME, we can reframe these obstacles as blueprints for next-generation strategies.

### From target validation to clinical conundrum: lessons from prototypic cancers

Lung, breast, and gastrointestinal cancers serve as paradigmatic models that collectively expose the core translational bottlenecks in SLC-targeted therapy. In NSCLC, the hypoxia and oncogene-driven overexpression of transporters like GLUT1 and MCT4 creates a clear therapeutic rationale. However, clinical attempts at inhibition often encounter rapid metabolic adaptation, where tumors augment oxidative phosphorylation or scavenge alternative nutrients [373]. This underscores a fundamental lesson: monotherapy targeting a single nutrient uptake route is insufficient due to inherent metabolic plasticity. Breast cancer heterogeneity further refines this principle. The stark dependence of TNBC on amino acid transporters like LAT1, contrasted with the flexibility of luminal subtypes, highlights that SLC dependencies are lineage- and driver-defined. This necessitates biomarker-driven patient selection, beyond which lies the challenge of on-target toxicity against widely expressed SLCs in normal tissues, narrowing the therapeutic window [97]. Perhaps the most complex lesson comes from gastrointestinal cancer. PDAC, thriving in a nutrient-poor, desmoplastic stroma, exemplifies the drug delivery barrier. Early trials combining SGLT2 inhibitors with chemotherapy show tolerability but variable efficacy, pointing to the hurdle of stromal exclusion [374]. Together, these cases converge on a unifying paradigm: The primary translational bottleneck is not target validity, but context dependency. This dependency is woven from three intertwined threads: (1) cell-intrinsic metabolic flexibility, (2) tumor-type-specific transcriptional and epigenetic programs, and (3) the physical and biochemical constraints of the TME.

### Beyond inhibition: next-generation strategies and mechanistic nuances

To overcome the challenges of redundancy, adaptation, and delivery, the field is evolving beyond simple pharmacological inhibition. Dual or poly-SLC targeting strategies aim to preempt compensatory pathways. For targets where catalytic inhibition is difficult, PROTAC-based degraders offer a powerful alternative, potentially achieving more durable suppression of overexpressed transporters like those in MYC-driven TNBC.

Crucially, the SLC superfamily includes key drug transporters themselves, a point vital for a comprehensive discussion on therapy. Families such as SLC22 (OCTs) and SLCO (OATPs) mediate the uptake of chemotherapeutics and TKIs. Their expression and polymorphism status can dictate tumor response or resistance, representing a direct link between SLC biology and established cancer therapies [375]. Similarly, bile acid transporters (e.g., ASBT/SLC10A2) are being exploited for tumor-selective drug delivery in liver and colorectal cancers, illustrating how SLCs can be leveraged as conduits rather than just targets [376–378].

Drug delivery challenges, particularly in stromal-rich or metastatic sites, are being addressed by nanoparticle and prodrug platforms engineered for SLC-mediated uptake. Glucose- or amino acid-conjugated nanoparticles can exploit the heightened metabolic appetite of tumors, improving selectivity and penetration.

Underpinning these strategies is a deepening understanding of SLC regulation. Beyond well-characterized transcriptional control by HIF-1α and MYC, epigenetic and post-translational layers add critical complexity. DNA methylation can silence tumor-suppressive SLCs, while histone modifications activate others. Post-translationally, phosphorylation, ubiquitination, and glycosylation dynamically regulate transporter trafficking, stability, and activity [162, 379, 380]. Targeting these regulatory nodes offers an alternative to direct transporter inhibition, potentially achieving more precise control over specific oncogenic SLC functions.

### Toward precision metabolic oncology: future directions and unresolved questions

The future of SLC-targeted therapy lies in mechanism-informed combination regimens rather than monotherapies. The most promising integrations are those that address reciprocal vulnerabilities. For example, combining SLC inhibitors with immunotherapy is supported by a clear mechanistic rationale: reducing lactate export via MCT4 inhibition can alleviate acidosis-driven suppression of T-cell function and promote a favorable immune phenotype [218]. Similarly, inhibiting the cystine-glutamate antiporter SLC7A11 can induce ferroptosis, an immunogenic form of cell death that may synergize with ICB [381].

To realize this potential, several frontiers must be advanced. First, mapping the “SLC context” requires high-resolution tools. Spatial metabolomics coupled with single-cell transcriptomics can define which SLCs are active in which tumor subpopulations and stromal cells within the TME. Second, developing dynamic biomarkers is essential. Circulating metabolites or extracellular vesicles carrying SLC proteins may provide real-time, non-invasive readouts of target engagement and emergent resistance. Third, the clinical pipeline must mature. Updated analysis of ongoing trials (e.g., NCT04542291 for SGLT2 inhibitors) should critically assess not just efficacy, but also pharmacodynamic biomarker correlates and mechanisms of failure [345].

In conclusion, SLC transporters are not merely passive nutrient gates; they are dynamic, context-dependent regulators of tumor fate and immune function. Their successful therapeutic integration mandates a shift from a “one-target-fits-all” approach to a precision metabolic oncology paradigm. This paradigm views SLCs as actionable vulnerabilities only within a defined biological context—a context shaped by tumor genetics, tissue lineage, microenvironmental pressures, and immune activity. By embracing this complexity, the field can move towards rational combinations that simultaneously starve tumors, remodel the TME, and enable anti-tumor immunity, ultimately delivering on the long-held promise of targeting cancer metabolism.

## Electronic supplementary material

Below is the link to the electronic supplementary material.

Supplementary material 1

## Data Availability

Not applicable.

## Abbreviations

SLC: Solute carrier
TME: Tumor microenvironment
MFS: Major facilitator superfamily
LeuT: Leucine transporter
DCs: Dendritic cells
TAMs: Tumor associated macrophages
CAFs: Cancer-associated fibroblasts
HCC: Hepatocellular carcinoma
CRC: Colorectal cancer
CYGB: Cytoglobin
GC: Gastric cancer
OBHS: Oxabicycloheptene sulfonate
TKI: Tyrosine kinase inhibitor
TMZ: Temozolomide
CTCs: Circulating tumor cells
SGLTs: Sodium-dependent glucose transporters
PDAC: Pancreatic ductal adenocarcinoma
EAAT1: Excitatory amino acid transporter 1
GSCs: Glioblastoma stem cells
HSC: Hematopoietic stem cell
LAT2: L-type amino acid transporter 2
FATPs: Fatty acid transport proteins
FAT: Fatty acid translocase
OXPHOS: Oxidation–oxidative phosphorylation
TNBC: Triple-negative breast cancer
MUFAs: Monounsaturated fatty acids
MDSCs: Myeloid-derived suppressor cells
ICB: Immune checkpoint blockade
ROS: Reactive oxygen species
G-MDSCs: Granulocytic myeloid-derived suppressor cells
ILC2: Innate lymphoid cell type 2 (ILC2)
cDC1s: Type-1 conventional dendritic cells
AHR: Aryl hydrocarbon receptor
PMN-MSDCs: Polymorphonuclear myeloid-derived suppressor cells
GlyCAF: Glycolytic CAF
PHB: Prohibitin-1
ANX2: Annexin 2
POSTN: Periostin
EPCs: Endothelial progenitor cells
MEPs: Megakaryocytic-erythroid progenitors
hENT1: Human equilibrative nucleoside transporter 1
hCNT3: Human concentrative nucleoside transporter 3
HDAC7: Histone deacetylase 7
OATPs: Organic anion transporting polypeptides
PDX: Patient-derived xenografts
LUAD: Lung adenocarcinoma
GTACs: GLUT-targeting chimeras
