# Supplementary material for: Solute Carrier transporters in tumor metabolism and immune modulation: implications for therapy
Source: J Transl Med. 2026 Mar 14;24:569. doi: 10.1186/s12967-026-07918-4 (PMC13101115; doi:10.1186/s12967-026-07918-4)
Supplement: Supplementary file 1 — Supplementary material 1 [file 12967_2026_7918_MOESM1_ESM.docx]

**Supplementary Table 1.** Summary of the regulation, signaling pathways, and therapeutic implications of key SLC transporters in cancer.

| Gene | Protein | Substrate | Cell Type | Upstream Regulation | Post-transcriptional and post-translational mechanisms | Downstream Signaling | Therapeutic Implication | Representative Cancer | Reference |
| --- | --- | --- | --- | --- | --- | --- | --- | --- | --- |
| SLC2A1 | GLUT1 | glucose | tumor | super-enhancers; YAP1–TEAD1; FOXM1; cMyc; Akt; mTOR; c-Jun | m6A; S-palmitoylation; SUMOylation; | GLUT1–mTORC1–HIF-1α–PKM2 positive feedback loop; | cell proliferation; cell migration and metastasis; PD-L1 expression | bladder cancer; lung adenocarcinoma | [1, 2] |
|  |  |  | macrophage | PERK–ATF4 |  |  | antitumor immunity in glioblastoma | glioblastoma | [3] |
|  |  |  | GlyCAF |  |  | CXCL6 | impede cytotoxic T cell infiltration | soft-tissue sarcomas | [4] |
| SLC2A2 | GLUT2 | glucose | tumor | HIF-1α; p38γ–PFKFB3 axis |  |  | tumor growth | KRAS-mutant cancers | [5] |
| SLC2A3 | GLUT3 | glucose | tumor | Cav1; AMPK/CREB1; RIP140; ZEB1; YTHDC1; ATF4/DDIT4 |  |  | TKI resistance; TMZ resistance; | TKI resistant tumor; glioblastoma | [6-9] |
| SLC2A4 | GLUT4 | glucose | tumor | KLF8; ISL1; AMPKα2/HNF4A/BORIS axis; | ALKBH5-mediated m6A demethylation |  | Proliferation; invasion and metastasis | HCC; breast cancer | [10-13] |
| SLC3A2 | CD98 | arachidonic acid | tumor |  |  |  | TAM M2 polarization | lung adenocarcinoma | [14] |
| SLC16A1 | MCT1 | lactate | tumor | SETDB1; MYC |  |  | tumor growth and metastasis | retinoblastoma; multiple myeloma | [15, 16] |
|  |  |  | terminally exhausted T cell |  |  |  | effector function | pan-cancer | [17] |
|  |  |  | Treg |  |  | NFAT1 | PD1 expresssion | low-glucose TME | [18] |
|  |  |  | neutrophil |  |  | NF-κB–COX-2 | T cell cytotoxicity | HCC | [19] |
| SLC16A3 | MCT4 | lactate | tumor |  | SYVN1-mediated ubiquitination |  | tumor growth | NSCLC | [20] |
| SLC5A1 | SGLT1 | glucose | tumor | PKCδ/EGFR axis |  |  | acquired resistance to EGFR TKIs | NSCLC | [21] |
| SLC1A3 | EAAT1 | glutamate | tumor | p53 |  |  | tumor growth and metastasis | T-cell acute lymphoblastic leukemia | [22-24] |
| SLC1A5 | ASCT2 | glutamine | tumor | lncRNA (LINC00857); MYC; |  |  | tumor proliferation, invasion; proteasome inhibitors resistance | glioma; HNSCC; CRC; multiple myeloma | [25]. |
| SLC1A6 | ASCT3 | glutamine | myeloid cell |  |  | IRE1α–XBP1 | accumulation of immunosuppressive GPR109A⁺ myeloid populations | liver cancer | [26] |
| SLC7A2 | CAT2 | lysine, arginine | tumor | RIOK3 |  | mTORC1 | invasion and metastasis | PDAC; glioblastoma | [27] |
| SLC7A3 | CAT3 | arginine | tumor | SP1 |  |  | cell migration | osteosarcoma | [28] |
| SLC7A5 | LAT1 | neutral amino acids | tumor | ATF4; mutant p53; circARID1A–IGF2BP; KRAS activation |  |  | tumor proliferation | CRC; GC | [29-32] |
|  |  |  | ILC2 |  |  |  | cellular expansion and cytokine production | helminth infection model | [33] |
|  |  |  | NK |  |  | c-Myc | metabolic reprogramming and effector function |  | [34] |
|  |  |  | CD4 T | c-Myc |  |  | dysfunctional differentiation states | autoimmunity model | [35] |
|  |  |  | endothelial | FOXC1 |  |  | angiogenesis and blood–retina barrier formation | retinal vascular disease | [36] |
| SLC7A8 | LAT2 | neutral amino acids | tumor |  |  | mTORC1–c-Myc | growth and metastasis | osteosarcoma | [37] |
| SLC7A11 | xCT | cystine | tumor | HMGA1; ATF4 | ALKBH5; OTUB1; USP52; O-GlcNAcylation; palmitoylation (ZDHHC8) |  | chemoresistance; ferroptosis supression | CRC; NSCLC; ESCC; | [38-40] |
| SLC27A1 | FATP1 | fatty acid | CD8⁺ T cell |  |  |  | mitochondrial dysfunction and impaired antitumor activity | multiple myeloma | [41] |
| SLC27A2 | FATP2 | fatty acid | tumor |  |  | PI3K–Akt–mTOR | proliferation and migration | bladder cancer | [42] |
|  |  |  | PMN-MDSC | RIPK3 |  |  | lipid uptake–driven immunosuppression | bladder cancer | [43] |
| CD36 | FAT | fatty acid | tumor |  | O-GlcNAcylation; | AKT/GSK-3β/β-catenin; Src–ERK; Src–PI3K–AKT–mTOR | migration and metastasis | gastric cancer; cervical cancer; HCC | [44-46] |
|  |  |  | CD8⁺ T cell |  |  |  | lipid peroxidation and ferroptosis | pan-cancer | [47] |
|  |  |  | Treg |  |  | PPAR-β | thrive in the lactate-rich TME | pan-cancer | [48] |
|  |  |  | endothelial | LPA/PKD-1-FoxO1 |  |  | proangiogenic reprogramming in the tumor vasculature | microvascular endothelial cells | [49] |
| SLC39A6 | LIV1 | zinc | tumor |  |  |  | metastasis | liver cancer | [50] |
| SLC6A3 | DAT | dopamine | tumor |  |  | histone methyltransferase G9a | tumor envasion | colon tumor | [51] |

**Reference:**

1. Fu, Z;Deng, M;Zhou, Q;Li, S;Liu, W;Cao, S, et al. Arsenic activated GLUT1-mTORC1/HIF-1α-PKM2 positive feedback networks promote proliferation and migration of bladder epithelial cells. The Science of the total environment. 2024:174538.

2. Zhou, J;Wang, D;Tang, D, Huang, W. Abnormal Activations of Super-Enhancers Enhance the Carcinogenicity in Lung Adenocarcinoma. Cancer management and research. 2020;12:8509-8518.

3. De Leo, A;Ugolini, A;Yu, X;Scirocchi, F;Scocozza, D;Peixoto, B, et al. Glucose-driven histone lactylation promotes the immunosuppressive activity of monocyte-derived macrophages in glioblastoma. Immunity. 2024;57(5):1105-1123.e1108.

4. Broz, M T;Ko, E Y;Ishaya, K;Xiao, J;De Simone, M;Hoi, X P, et al. Metabolic targeting of cancer associated fibroblasts overcomes T-cell exclusion and chemoresistance in soft-tissue sarcomas. Nature communications. 2024;15(1):2498.

5. Wang, F;Qi, X M;Wertz, R;Mortensen, M;Hagen, C;Evans, J, et al. p38γ MAPK Is Essential for Aerobic Glycolysis and Pancreatic Tumorigenesis. Cancer research. 2020;80(16):3251-3264.

6. Simpson, I A;Dwyer, D;Malide, D;Moley, K H;Travis, A, Vannucci, S J. The facilitative glucose transporter GLUT3: 20 years of distinction. American journal of physiology Endocrinology and metabolism. 2008;295(2):E242-253.

7. Ali, A;Levantini, E;Fhu, C W;Teo, J T;Clohessy, J G;Goggi, J L, et al. CAV1 - GLUT3 signaling is important for cellular energy and can be targeted by Atorvastatin in Non-Small Cell Lung Cancer. Theranostics. 2019;9(21):6157-6174.

8. Dai, W;Xu, Y;Mo, S;Li, Q;Yu, J;Wang, R, et al. GLUT3 induced by AMPK/CREB1 axis is key for withstanding energy stress and augments the efficacy of current colorectal cancer therapies. Signal transduction and targeted therapy. 2020;5(1):177.

9. Yan, B;Li, X;Peng, M;Zuo, Y;Wang, Y;Liu, P, et al. The YTHDC1/GLUT3/RNF183 axis forms a positive feedback loop that modulates glucose metabolism and bladder cancer progression. Experimental & molecular medicine. 2023;55(6):1145-1158.

10. Mao, A;Zhou, X;Liu, Y;Ding, J;Miao, A, Pan, G. KLF8 is associated with poor prognosis and regulates glycolysis by targeting GLUT4 in gastric cancer. Journal of cellular and molecular medicine. 2019;23(8):5087-5097.

11. Guo, T;Bai, Y H;Cheng, X J;Han, H B;Du, H;Hu, Y, et al. Insulin gene enhancer protein 1 mediates glycolysis and tumorigenesis of gastric cancer through regulating glucose transporter 4. Cancer communications (London, England). 2021;41(3):258-272.

12. Huang, Y;Xian, L;Liu, Z;Wei, L;Qin, L;Xiong, Y, et al. AMPKα2/HNF4A/BORIS/GLUT4 pathway promotes hepatocellular carcinoma cell invasion and metastasis in low glucose microenviroment. Biochemical pharmacology. 2022;203:115198.

13. Liu, H;Lyu, H;Jiang, G;Chen, D;Ruan, S;Liu, S, et al. ALKBH5-Mediated m6A Demethylation of GLUT4 mRNA Promotes Glycolysis and Resistance to HER2-Targeted Therapy in Breast Cancer. Cancer research. 2022;82(21):3974-3986.

14. Li, Z;Chen, S;He, X;Gong, S;Sun, L, Weng, L. SLC3A2 promotes tumor-associated macrophage polarization through metabolic reprogramming in lung cancer. Cancer science. 2023;114(6):2306-2317.

15. She, X;Wu, Q;Rao, Z;Song, D;Huang, C;Feng, S, et al. SETDB1 Methylates MCT1 Promoting Tumor Progression by Enhancing the Lactate Shuttle. Advanced science (Weinheim, Baden-Wurttemberg, Germany). 2023;10(28):e2301871.

16. Liu, X;Qin, H;Zhang, L;Jia, C;Chao, Z;Qin, X, et al. Hyperoxia induces glucose metabolism reprogramming and intracellular acidification by suppressing MYC/MCT1 axis in lung cancer. Redox biology. 2023;61:102647.

17. Peralta, R M;Xie, B;Lontos, K;Nieves-Rosado, H;Spahr, K;Joshi, S, et al. Dysfunction of exhausted T cells is enforced by MCT11-mediated lactate metabolism. Nature immunology. 2024.

18. Kumagai, S;Koyama, S;Itahashi, K;Tanegashima, T;Lin, Y T;Togashi, Y, et al. Lactic acid promotes PD-1 expression in regulatory T cells in highly glycolytic tumor microenvironments. Cancer cell. 2022;40(2):201-218.e209.

19. Deng, H;Kan, A;Lyu, N;He, M;Huang, X;Qiao, S, et al. Tumor-derived lactate inhibit the efficacy of lenvatinib through regulating PD-L1 expression on neutrophil in hepatocellular carcinoma. Journal for immunotherapy of cancer. 2021;9(6).

20. Zhao, M;Huang, C;Yang, L;Pan, B;Yang, S;Chang, J, et al. SYVN1-mediated ubiquitylation directs localization of MCT4 in the plasma membrane to promote the progression of lung adenocarcinoma. Cell death & disease. 2023;14(10):666.

21. Chen, C H;Wang, B W;Hsiao, Y C;Wu, C Y;Cheng, F J;Hsia, T C, et al. PKCδ-mediated SGLT1 upregulation confers the acquired resistance of NSCLC to EGFR TKIs. Oncogene. 2021;40(29):4796-4808.

22. Tajan, M;Hock, A K;Blagih, J;Robertson, N A;Labuschagne, C F;Kruiswijk, F, et al. A Role for p53 in the Adaptation to Glutamine Starvation through the Expression of SLC1A3. Cell Metab. 2018;28(5):721-736.e726.

23. Garcia-Bermudez, J;Baudrier, L;La, K;Zhu, X G;Fidelin, J;Sviderskiy, V O, et al. Aspartate is a limiting metabolite for cancer cell proliferation under hypoxia and in tumours. Nature cell biology. 2018;20(7):775-781.

24. Sun, J;Nagel, R;Zaal, E A;Ugalde, A P;Han, R;Proost, N, et al. SLC1A3 contributes to L-asparaginase resistance in solid tumors. The EMBO journal. 2019;38(21):e102147.

25. Shen, Q;Wang, R;Liu, X;Song, P;Zheng, M;Ren, X, et al. HSF1 Stimulates Glutamine Transport by Super-Enhancer-Driven lncRNA LINC00857 in Colorectal Cancer. Cancers. 2022;14(16).

26. Yang, Y;Pei, T;Liu, C;Cao, M;Hu, X;Yuan, J, et al. Glutamine metabolic competition drives immunosuppressive reprogramming of intratumour GPR109A(+) myeloid cells to promote liver cancer progression. Gut. 2024.

27. Qin, H;Sun, R;Guo, X;Fang, L;Xu, M;Teng, Y, et al. RIOK3 promotes mTORC1 activation by facilitating SLC7A2-mediated arginine uptake in pancreatic ductal adenocarcinoma. Aging. 2023;15(4):1039-1051.

28. Wang, P;Song, Y;Li, H;Zhuang, J;Shen, X;Yang, W, et al. SIRPA enhances osteosarcoma metastasis by stabilizing SP1 and promoting SLC7A3-mediated arginine uptake. Cancer letters. 2023;576:216412.

29. Martinez, R S;Salji, M J;Rushworth, L;Ntala, C;Rodriguez Blanco, G;Hedley, A, et al. SLFN5 Regulates LAT1-Mediated mTOR Activation in Castration-Resistant Prostate Cancer. Cancer research. 2021;81(13):3664-3678.

30. Tombari, C;Zannini, A;Bertolio, R;Pedretti, S;Audano, M;Triboli, L, et al. Mutant p53 sustains serine-glycine synthesis and essential amino acids intake promoting breast cancer growth. Nature communications. 2023;14(1):6777.

31. Ma, Q;Yang, F;Huang, B;Pan, X;Li, W;Yu, T, et al. CircARID1A binds to IGF2BP3 in gastric cancer and promotes cancer proliferation by forming a circARID1A-IGF2BP3-SLC7A5 RNA-protein ternary complex. Journal of experimental & clinical cancer research : CR. 2022;41(1):251.

32. Najumudeen, A K;Ceteci, F;Fey, S K;Hamm, G;Steven, R T;Hall, H, et al. The amino acid transporter SLC7A5 is required for efficient growth of KRAS-mutant colorectal cancer. Nature genetics. 2021;53(1):16-26.

33. Hodge, S H;Krauss, M Z;Kaymak, I;King, J I;Howden, A J M;Panic, G, et al. Amino acid availability acts as a metabolic rheostat to determine the magnitude of ILC2 responses. The Journal of experimental medicine. 2023;220(3).

34. Loftus, R M;Assmann, N;Kedia-Mehta, N;O'Brien, K L;Garcia, A;Gillespie, C, et al. Amino acid-dependent cMyc expression is essential for NK cell metabolic and functional responses in mice. Nature communications. 2018;9(1):2341.

35. Zhang, W;Cao, X;Zhong, X;Wu, H;Shi, Y;Feng, M, et al. SRC2 controls CD4(+) T cell activation via stimulating c-Myc-mediated upregulation of amino acid transporter Slc7a5. Proceedings of the National Academy of Sciences of the United States of America. 2023;120(18):e2221352120.

36. Bhakuni, T;Norden, P R;Ujiie, N;Tan, C;Lee, S K;Tedeschi, T, et al. FOXC1 regulates endothelial CD98 (LAT1/4F2hc) expression in retinal angiogenesis and blood-retina barrier formation. Nature communications. 2024;15(1):4097.

37. Wang, Z;Li, B;Li, S;Lin, W;Wang, Z;Wang, S, et al. Metabolic control of CD47 expression through LAT2-mediated amino acid uptake promotes tumor immune evasion. Nature communications. 2022;13(1):6308.

38. Yang, J Y;Lei, X Y;He, K Y;Guo, J R;Liu, M J;Li, J Q, et al. HMGA1 drives chemoresistance in esophageal squamous cell carcinoma by suppressing ferroptosis. Cell death & disease. 2024;15(2):158.

39. Luo, J;Yu, H;Yuan, Z;Ye, T, Hu, B. ALKBH5 decreases SLC7A11 expression by erasing m6A modification and promotes the ferroptosis of colorectal cancer cells. Clinical & translational oncology : official publication of the Federation of Spanish Oncology Societies and of the National Cancer Institute of Mexico. 2023;25(7):2265-2276.

40. Huang, Z;Lin, G;Hong, Y;Weng, L;Zhu, K, Zhuang, W. High expression of AlkB homolog 5 suppresses the progression of non-small cell lung cancer by facilitating ferroptosis through m6A demethylation of SLC7A11. Environ Toxicol. 2024;39(7):4035-4046.

41. Gudgeon, N;Giles, H;Bishop, E L;Fulton-Ward, T;Escribano-Gonzalez, C;Munford, H, et al. Uptake of long-chain fatty acids from the bone marrow suppresses CD8+ T-cell metabolism and function in multiple myeloma. Blood advances. 2023;7(20):6035-6047.

42. Jiang, M;Chen, R;Hu, B;Xiong, S;Li, S;Fu, B, et al. FATP2 activates PI3K/Akt/mTOR pathway by inhibiting ATF3 and promotes the occurrence and development of bladder cancer. Cellular signalling. 2024;117:111087.

43. Veglia, F;Tyurin, V A;Blasi, M;De Leo, A;Kossenkov, A V;Donthireddy, L, et al. Fatty acid transport protein 2 reprograms neutrophils in cancer. Nature. 2019;569(7754):73-78.

44. Pan, J;Fan, Z;Wang, Z;Dai, Q;Xiang, Z;Yuan, F, et al. CD36 mediates palmitate acid-induced metastasis of gastric cancer via AKT/GSK-3β/β-catenin pathway. Journal of experimental & clinical cancer research : CR. 2019;38(1):52.

45. Yang, P;Su, C;Luo, X;Zeng, H;Zhao, L;Wei, L, et al. Dietary oleic acid-induced CD36 promotes cervical cancer cell growth and metastasis via up-regulation Src/ERK pathway. Cancer letters. 2018;438:76-85.

46. Luo, X;Zheng, E;Wei, L;Zeng, H;Qin, H;Zhang, X, et al. The fatty acid receptor CD36 promotes HCC progression through activating Src/PI3K/AKT axis-dependent aerobic glycolysis. Cell death & disease. 2021;12(4):328.

47. Ma, X;Xiao, L;Liu, L;Ye, L;Su, P;Bi, E, et al. CD36-mediated ferroptosis dampens intratumoral CD8(+) T cell effector function and impairs their antitumor ability. Cell Metab. 2021;33(5):1001-1012.e1005.

48. Wang, H;Franco, F;Tsui, Y C;Xie, X;Trefny, M P;Zappasodi, R, et al. CD36-mediated metabolic adaptation supports regulatory T cell survival and function in tumors. Nature immunology. 2020;21(3):298-308.

49. Ren, B;Best, B;Ramakrishnan, D P;Walcott, B P;Storz, P, Silverstein, R L. LPA/PKD-1-FoxO1 Signaling Axis Mediates Endothelial Cell CD36 Transcriptional Repression and Proangiogenic and Proarteriogenic Reprogramming. Arteriosclerosis, thrombosis, and vascular biology. 2016;36(6):1197-1208.

50. Yu, Z;Chen, C;Gu, H;Dong, J;Zhang, Y;Wang, J, et al. Suppression of SLC39A6-CREB1 axis in liver cancer causes PCK1-mediated mitochondrial dysfunction. Cell proliferation. 2023;56(10):e13527.

51. Bergin, C J;Zouggar, A;Mendes da Silva, A;Fenouil, T;Haebe, J R;Masibag, A N, et al. The dopamine transporter antagonist vanoxerine inhibits G9a and suppresses cancer stem cell functions in colon tumors. Nature cancer. 2024;5(3):463-480.
